# Environmental Controls of Oyster-Pathogenic Vibrio spp. in Oregon Estuaries and a Shellfish Hatchery

**DOI:** 10.1128/AEM.02156-17

**Published:** 2018-04-16

**Authors:** Mary R. Gradoville, Byron C. Crump, Claudia C. Häse, Angelicque E. White

**Affiliations:** a College of Earth, Ocean, and Atmospheric Sciences, Oregon State University, Corvallis, Oregon, USA; b Department of Biomedical Sciences, College of Veterinary Medicine, Oregon State University, Corvallis, Oregon, USA; Rutgers, The State University of New Jersey

**Keywords:** 16S rRNA, Vibrio, environmental pathogens, estuarine ecology

## Abstract

Vibrio spp. have been a persistent concern for coastal bivalve hatcheries, which are vulnerable to environmental pathogens in the seawater used for rearing larvae, yet the biogeochemical drivers of oyster-pathogenic Vibrio spp. in their planktonic state are poorly understood. Here, we present data tracking oyster-pathogenic Vibrio bacteria in Netarts Bay and Yaquina Bay in Oregon, USA, as well as in adjacent coastal waters and a local shellfish hatchery, through the 2015 upwelling season. Vibrio populations were quantified using a culture-independent approach of high-throughput Vibrio-specific 16S rRNA gene sequencing paired with droplet digital PCR, and abundances were analyzed in the context of local biogeochemistry. The most abundant putative pathogen in our samples was Vibrio coralliilyticus. Environmental concentrations of total Vibrio spp. and V. coralliilyticus were highest in Netarts Bay sediment samples and higher in seawater from Netarts Bay than from nearshore coastal waters or Yaquina Bay. In Netarts Bay, the highest V. coralliilyticus concentrations were observed during low tide, and abundances increased throughout the summer. We hypothesize that the warm shallow waters in estuarine mudflats facilitate the local growth of the V. coralliilyticus pathogen. Samples from larval oyster tanks in Whiskey Creek Shellfish Hatchery, which uses seawater pumped directly from Netarts Bay, contained significantly lower total Vibrio species concentrations, but roughly similar V. coralliilyticus concentrations, than did the bay water, resulting in a 30-fold increase in the relative abundance of the V. coralliilyticus pathogen in hatchery tanks. This suggests that the V. coralliilyticus pathogen is able to grow or persist under hatchery conditions.

**IMPORTANCE** It has been argued that oyster-pathogenic Vibrio spp. have contributed to recent mortality events in U.S. shellfish hatcheries (R. A. Elston, H. Hasegawa, K. L. Humphrey, I. K. Polyak, and C. Häse, Dis Aquat Organ 82:119–134, 2008, https://doi.org/10.3354/dao01982); however, these events are often sporadic and unpredictable. The success of hatcheries is critically linked to the chemical and biological composition of inflowing seawater resources; thus, it is pertinent to understand the biogeochemical drivers of oyster-pathogenic Vibrio spp. in their planktonic state. Here, we show that Netarts Bay, the location of a local hatchery, is enriched in oyster-pathogenic V. coralliilyticus compared to coastal seawater, and we hypothesize that conditions in tidal flats promote the local growth of this pathogen. Furthermore, V. coralliilyticus appears to persist in seawater pumped into the local hatchery. These results improve our understanding of the ecology and environmental controls of the V. coralliilyticus pathogen and could be used to improve future aquaculture efforts, as multiple stressors impact hatchery success.

## INTRODUCTION

The Vibrionaceae (which includes the genus Vibrio) are a genetically and ecologically diverse group of pathogenic and benign heterotrophic Gram-negative bacteria present in most, if not all, marine ecosystems (1, 2). Vibrionaceae include >130 described species (3) with diverse life histories. Populations of planktonic marine Vibrio spp. can survive in a dormant state under unfavorable conditions (4) but grow rapidly in response to temperature and nutrient pulses (5), aided by numerous ribosome genes (6). Additionally, many Vibrio species are associated with marine particles and/or living hosts, where they can act as mutual symbionts or disease agents. Vibrio species include several human pathogens, including V. cholerae (7, 8), V. parahaemolyticus, and V. vulnificus, as well as numerous pathogens of marine mammals, fish, and shellfish (9).

Pathogenic Vibrio spp. have been a historical concern for the aquaculture industry (10–12), and it has been argued that infections of V. coralliilyticus (formerly misclassified as V. tubiashii [13–15]) are also a potential threat to production in oyster hatcheries located on the northwest coast of the United States (16). These hatcheries use seawater pumped directly from coastal or estuarine waters with minimal treatment steps (i.e., sand filters and heating); thus, larval rearing success is critically linked to the chemical and biological composition of seawater resources. Indeed, hatchery production has been depressed in recent years, and while pathogenic Vibrio spp. were initially implicated (16), Barton et al. (17, 18) offered strong evidence that unfavorable carbonate chemistry (ocean acidification) was the more significant cause. Wind-driven coastal upwelling in summer months delivers waters to hatcheries that have a low aragonite saturation state, which is a carbonate system parameter that is mechanistically linked to larval growth and fitness under controlled conditions (19) and has been correlated with larval production in an Oregon hatchery (17). However, hatchery mortality events are sporadic and unpredictable, and determinations of the underlying mechanisms (acidification, pathogens, and/or other stressors) are often speculative. Thus, the possibility that V. coralliilyticus infections have played a role in hatchery mortality events cannot be excluded. Furthermore, while much recent attention has focused on monitoring the intrusion of acidified waters into coastal and estuarine environments (20, 21), it is not known whether the spatiotemporal distributions of oyster-pathogenic Vibrio spp. are linked to particular offshore water masses or to local chemical and biological conditions.

The abundance and community structure of coastal and estuarine Vibrio populations are shaped by environmental drivers (22–24). However, these patterns can be complex, as individual Vibrio species display different relationships with physical/chemical conditions (e.g., temperature and salinity) and with local biology, due in part to species-specific associations with hosts, including phytoplankton and zooplankton (9). Previous studies of V. coralliilyticus in coral reef systems have observed increased abundances associated with elevated temperatures (24, 25), but the ecology of this organism has rarely been assessed in temperate ecosystems. In the only previous study of V. coralliilyticus in the Northeast Pacific coastal upwelling zone, Elston et al. (16) hypothesized that V. coralliilyticus populations in Netarts Bay, OR were seeded via upwelling of nearshore waters, with subsequent local growth following relaxation of upwelling-favorable winds and warming. However, this study was qualitative in nature and relied on plating and culturing methods which bias the community structure of Vibrio species (4). Thus, the ecological controls of oyster-pathogenic Vibrio spp. in coastal upwelling-influenced systems, including estuaries containing larval hatcheries, remain poorly understood.

To this end, we designed a study to track the diversity and abundance of total and oyster-pathogenic Vibrio spp. in Oregon estuaries, adjacent coastal waters, and a larval oyster hatchery. In lieu of plating and culturing approaches, we used high-throughput Vibrio-specific 16S rRNA gene sequencing and droplet digital PCR to quantify Vibrio populations, and assessed pathogen abundances in the context of the biological, chemical, and physical metrics of the Vibrio habitat. The overarching aim of our study was to investigate the spatiotemporal patterns of oyster-pathogenic Vibrio populations in Oregon estuaries, as well as the environmental controls of the most dominant pathogen, V. coralliilyticus.

## RESULTS

### Environmental conditions.

**FIG 1:**
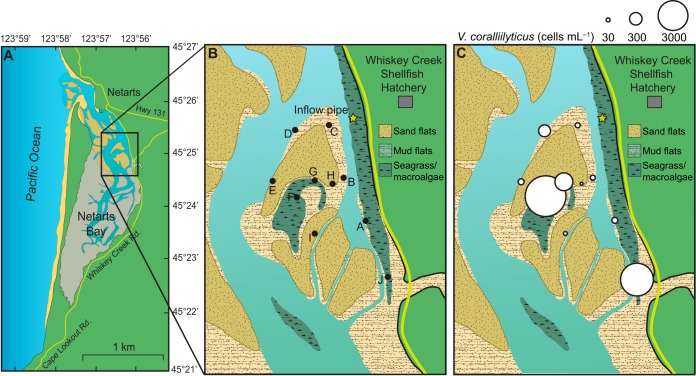
Map of Netarts Bay (A) showing locations of tidal flat sampling stations, as well as locations of the Whiskey Creek Shellfish Hatchery (WCSH) and the WCSH pipe inlet (B). (C) Average V. coralliilyticus concentrations at tidal flat sampling stations (sampled during low tide on 15 July, 30 July, and 29 August 2015). See Fig. S7 for the full tidal flat sampling data set.

Samples were collected through the 2015 coastal upwelling season (Table 1, Fig. 1), during which a wide range of biological and chemical conditions were encountered in Netarts Bay and Yaquina Bay and in vertical profiles of the water column at offshore stations. The ranges of temperature, salinity, chlorophyll, nutrients, partial pressure of carbon dioxide (P_CO2_), and daily wind stress are presented in Table S1 in the supplemental material for the estuarine and offshore sampling stations. Sampling spanned upwelling- and downwelling-favorable conditions (Table S1).

**TABLE 1:** Description of sampling stations used in this study

Location	Sample type(s)^*a*^	Description	Sampling time period	No. of days sampled	No. of DNA samples
Netarts WCSH intake	DNA, FCM, nutrients, Chl *a*, T, S, P_CO2_	Pipe in WCSH sampled directly from Netarts Bay (Fig. 1)	May–September 2015	13	62
Netarts Bay tidal flat	DNA (SW), DNA (sediment), FCM, nutrients, Chl *a*, T	Tidal channels, isolated seawater pools, seagrass beds, mudflats, and sand flats sampled at low tide (Fig. 1).	July–August 2015	3	56
WCSH larval tanks	DNA, FCM	Oyster larval rearing tanks at WCSH	May–September 2015	10	19
Yaquina Bay	DNA, FCM, nutrients, Chl *a*, T, S	OSU pumphouse dock; located near the mouth of Yaquina Bay (44.62°N, −124.04°W)	July–September 2015	6	22
Coastal NH10	DNA, FCM, nutrients, Chl *a*, T, S	Nearshore station; 80 m depth; located at 44.65°N, −124.29°W	October 2014	1	5
Coastal CE0405	DNA, FCM, nutrients, Chl *a*, T, S	Nearshore station; 588 m depth; located at 44.37°N, −124.95°W	October 2014	1	8
Coastal NH5	DNA, FCM, nutrients, Chl *a*, T, S, P_CO2_	Nearshore station; 59 m depth; located at 44.65°N, −124.18°W	September 2015	1	4
Coastal NH25	DNA, FCM, nutrients, Chl *a*, T, S, P_CO2_	Nearshore station; 293 m depth; located at 44.65°N, −124.65°W	September 2015	1	6

a FCM, flow cytometry.

### Diversity of Vibrio species populations and putative oyster-pathogenic Vibrio species.

Vibrio species diversity was assessed via high-throughput sequencing of a 491-bp region of the 16S rRNA gene, using primers specific to the family Vibrionaceae (see Materials and Methods). DNA from the cultured V. coralliilyticus strain RE22 was also sequenced as a positive control. Only samples with three successful PCR amplifications were used for sequencing (23/23 coastal seawater samples, 21/22 Yaquina Bay seawater samples, 30/30 Netarts tidal flat seawater samples, 18/28 Netarts tidal flat sediment samples, 58/62 Netarts Whiskey Creek Shellfish Hatchery [WCSH] intake seawater samples, and 9/19 WCSH larval tank samples). Subsampling down to 7,145 sequences per sample resulted in near saturation for most rarefaction curves (Fig. S1). Clustering sequences at 97% nucleotide identity resulted in 1,950 operational taxonomic units (OTUs) distributed among the Vibrionaceae species (Fig. S2).

**FIG 2:**
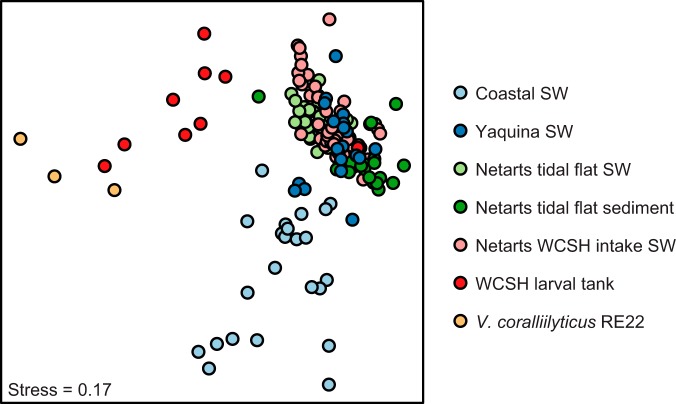
Nonmetric multidimensional scaling (NMDS) plot derived from the Bray-Curtis dissimilarity matrix of Vibrio species 16S rRNA OTUs clustered at 97% identity. Each point represents an individual sample. Colors represent sample type. Three replicate DNA samples from a V. coralliilyticus strain RE22 culture were sequenced and included for comparison. SW, seawater.

The community structure of Vibrio populations varied among the 7 sample types (Fig. 2) (global analysis of similarity [ANOSIM] *R* = 0.545, *P* = 0.001). Among environmental samples, the strongest differences in Vibrio community structure were observed between coastal seawater and the two types of seawater samples from Netarts Bay (ANOSIM *R* = 0.84 and 0.75, *P* = 0.001 for pairwise tests between coastal seawater samples and seawater from the Netarts Bay WCSH intake and Netarts Bay stations, respectively). In contrast, estuary samples from Netarts Bay and Yaquina Bay contained similar Vibrio community structure (ANOSIM pairwise tests, *R* < 0.5). Samples from WCSH larval tanks contained Vibrio communities which differed strongly from all environmental samples (ANOSIM *R* > 0.77, *P* = 0.001 for all pairwise tests), and were more similar to DNA samples from cultured V. coralliilyticus strain RE22 (Fig. 2) (ANOSIM pairwise test *R* = 0.36, *P* = 0.036). The taxonomic composition of Vibrio spp. also varied by sample type (Fig. S3). An indicator species analysis identified particular Vibrionaceae phylotypes associated with the environments sampled in our study. For example, the strongest indicator phylotype for coastal seawater samples clustered with Photobacterium aquimarinus (OTU 1687), while the strongest indicator phylotype for Netarts WCSH intake seawater samples clustered with Aliivibrio finisterrensis (OTU 2075, Table S2 and Fig. S2).

Putative oyster-pathogenic Vibrio spp. were classified based on inferred phylogenetic relationships among Vibrio OTUs and a representative set of 134 cultured Vibrio species (Fig. S2). Our phylogenetic tree contains a topology similar to that of a previous 16S rRNA-based tree in a review of the phylogeny of Vibrionaceae species (3); thus, we view our species designations as robust, despite the limitations of using 16S rRNA-based phylogeny for strain-level classifications of Vibrio species (3).

Our samples contained sequences clustering with the bivalve larval pathogens V. coralliilyticus, V. tubiashii, V. parahaemolyticus, V. pectenicida, and V. cholerae but did not contain sequences clustering with V. alginolyticus, V. splendidus, or V. vulnificus (Fig. S4). The most abundant putative-pathogenic species was V. coralliilyticus in all sample types, with the exception of Yaquina Bay seawater samples, where the most abundant putative pathogen was V. cholerae. Interestingly, 10 out of the 13 OTUs clustering with V. coralliilyticus were identified as indicator phylotypes for Netarts WCSH intake seawater samples, while the only OTU clustering with V. cholerae (OTU 30) was the strongest indicator phylotype for Yaquina Bay seawater samples. We focused remaining analyses on sequences classified as V. coralliilyticus.

### Abundances of Vibrio spp. and V. coralliilyticus.

**FIG 3:**
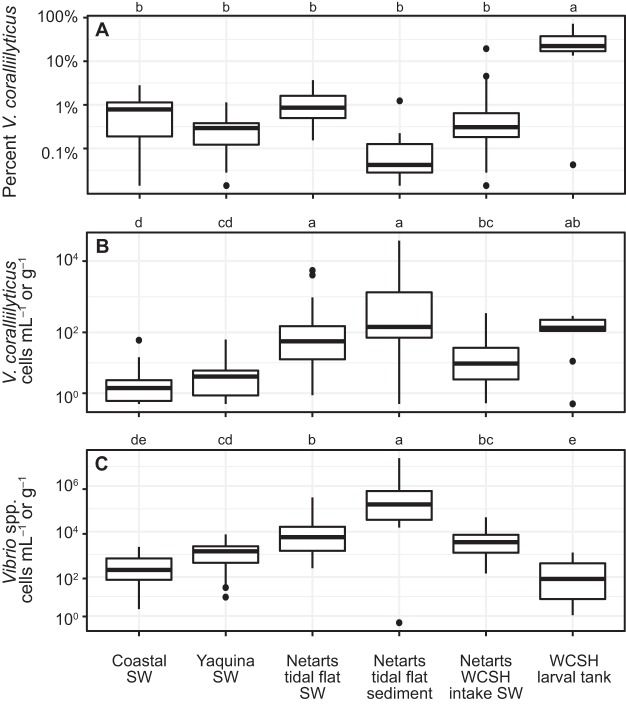
The percentage of total Vibrio spp. classified as V. coralliilyticus (A) and abundance estimates for V. coralliilyticus (B) and total Vibrio spp. (C). Concentrations are normalized to milliliter of seawater or to gram of sediment (Netarts sediment samples only). Letters above each panel note statistical significance, where different letters signify significant differences in percent (A) or log-transformed abundance (B and C) (Tukey HSD, *P* < 0.05), and categories with the same letter are not statistically different from one another. Boxplots represent medians as thick horizontal lines, 25 to 75% quantiles as boxes, the smallest and largest values (at most 1.5 times the interquartile range) as whiskers, and outliers as dots.

The fraction of total Vibrio sequences classified as V. coralliilyticus was higher in WCSH larval rearing tanks than in all other sample types (Fig. 3A and S3). V. coralliilyticus represented a mean of 26% of the Vibrio sequences in tank samples, a percentage which was 33-fold higher than in coastal seawater samples and 30-fold higher than in Netarts WCSH intake seawater samples (Tukey honestly significant difference [HSD], *P* < 0.0001 for both). Among environmental samples, the mean percentage of V. coralliilyticus sequences was lower in Netarts tidal flat sediment than in all seawater sample types, but these differences were not statistically significant (Tukey HSD, *P* > 0.05).

**FIG 4:**
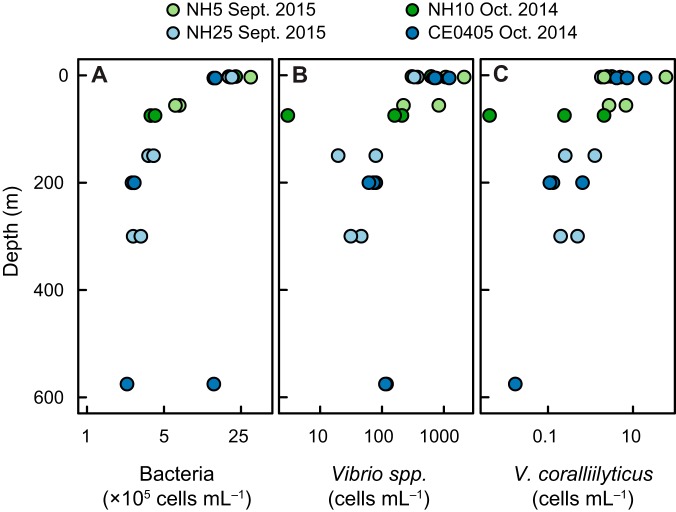
Abundances of heterotrophic bacteria (A), Vibrio spp. (B), and V. coralliilyticus (C) at shelf-break Oregon stations. See Table 1 for a description of sampling sites.

Relative abundances of V. coralliilyticus from 16S rRNA gene sequences were combined with droplet digital PCR (ddPCR)-derived concentrations of total Vibrio 16S rRNA gene copies to produce estimates for V. coralliilyticus cell abundance (Fig. 3). Total Vibrio species abundance estimates ranged from 1.1 × 10^1^ to 3.2 × 10^5^ cells · ml^−1^ across sample types. When normalizing to milliliters of seawater or grams of sediment, the highest concentrations of V. coralliilyticus were observed in Netarts Bay tidal flat station sediment samples, Netarts Bay tidal flat seawater samples, and WCSH larval tank samples (Fig. 3). Among seawater samples, V. coralliilyticus concentrations were lowest in coastal seawater and highest in the Netarts tidal flats. Total Vibrio species abundance estimates followed similar trends among sample types, excluding WCSH larval tank samples, which contained the lowest concentrations of total Vibrio species. Normalizing abundances to DNA content resulted in similar trends across sample types, with the exception of Netarts tidal flat sediment samples; these samples had the highest abundances of all sample types when normalized to sediment mass but the lowest abundances when normalized to DNA content, which was ∼3 orders of magnitude higher in sediment (per gram) than in seawater (per milliliter) (Fig. S5). At coastal stations, the abundances of Vibrio spp., V. coralliilyticus, and total heterotrophic bacteria all decreased with increasing depth (Fig. 4).

The abundances of V. coralliilyticus and total Vibrio spp. were highly variable. In Netarts Bay tidal flat seawater samples, the variability within biological replicates of Vibrio spp. and V. coralliilyticus was an order of magnitude greater than the biological variability observed in total heterotrophic bacterial abundance (assessed via flow cytometry) (Table S3). Likewise, variability between biological replicates of Vibrio spp. and V. coralliilyticus in Netarts WCSH intake seawater was an order of magnitude higher than that of total heterotrophic bacteria and was nearly equal in magnitude to the total daily variability (Table S3). Despite this large biological heterogeneity, there were significant differences in V. coralliilyticus concentrations among days for the Netarts WCSH intake time series (one-way analysis of variance [ANOVA], *P* < 0.001; Fig. S6) and marginally significant differences among Netarts tidal flat stations (one-way ANOVA *P* = 0.08; Fig. 1 and S7). Additionally, V. coralliilyticus concentrations had a larger total range (3 orders of magnitude in Netarts WCSH intake water) and higher day-to-day variability than concentrations of total heterotrophic bacteria.

### Environmental predictors of Vibrio spp. and V. coralliilyticus.

Despite the high variability observed within biological replicates, regression models showed evidence for environmental predictors of V. coralliilyticus and Vibrio species concentrations. Concentrations of both groups were positively correlated with the day of year (Fig. S6, linear regression *P* < 0.001, *R*^2^ = 0.26 and 0.22 for Vibrio spp. and V. coralliilyticus, respectively), suggesting a seasonal cycle in Vibrio growth. Models testing environmental explanatory variables indicate that both V. coralliilyticus and total Vibrio spp. were negatively correlated with northward downwelling-favorable wind stress and positively correlated with the phosphate concentration (Table 2). Additionally, total Vibrio species concentrations were positively correlated with temperature, while V. coralliilyticus concentrations were negatively correlated with high tide. Furthermore, tide, wind stress, and phosphate were also significant explanatory variables predicting the ratio of V. coralliilyticus to heterotrophic bacteria, indicating that these variables help to explain how patterns of V. coralliilyticus diverge from the average bacterioplankton, whose abundances were negatively related to the concentration of nitrate plus nitrite and positively related to temperature and the concentration of phosphate (Table 2).

**TABLE 2:** Results from linear regression models testing for environmental predictors of V. coralliilyticus, Vibrio spp., heterotrophic bacteria, and the ratio of V. coralliilyticus to total heterotrophic bacteria in the Netarts WCSH intake seawater samples

Response variable^*a*^	Explanatory variables^*b*^											
	β1				β2				β3			
	β1	Sign	*R*^2^	*P* value	β2	Sign	*R*^2^	*P* value	β3	Sign	*R*^2^	*P* value
Vibrio cells · ml^−1^	Temp	+	0.26	<0.001	Wind	−	0.14	0.001	PO_4_	+	0.11	0.04
V. coralliilyticus cells · ml^−1^	PO_4_	+	0.25	<0.001	Wind	−	0.10	0.04	Tide	−	0.10	0.04
V. coralliilyticus-to-heterotrophic bacteria ratio	PO_4_	+	0.23	<0.001	Wind	−	0.13	0.01	Tide	−	0.09	0.008
Heterotrophic bacteria cells · ml^−1^	PO_4_	+	0.06	0.02	N+N	−	0.27	<0.001	Temp	+	0.29	0.009

a Note that all response variables (excluding the ratio of V. coralliilyticus to total heterotrophic bacteria) were log-transformed prior to regression analyses.

b Statistically significant explanatory variables (β: phosphate [PO_4_], daily wind stress [wind], nitrate plus nitrite [N+N], discrete tidal height [tide, in meters], and temperature [temp]), associated *P* values, and relative contributions of each explanatory variable to the overall *R*^2^ value from four separate linear regression models (rows).

## DISCUSSION

Environmental pathogens, such as Vibrio spp., act as disease agents for a variety of hosts, including shellfish, but can also persist and thrive in marine environments independently (26). Understanding the ecology of these organisms in their planktonic state can help determine environmental controls of marine disease and improve aquaculture efforts. In this study, we used a culture-independent molecular approach to investigate the diversity and spatiotemporal abundance patterns of oyster-pathogenic Vibrio spp. in Oregon estuaries, coastal seawater, and a larval oyster hatchery. The most abundant putative pathogen in our samples clustered with V. coralliilyticus, a known oyster pathogen (27). V. coralliilyticus concentrations were highest in samples from Netarts Bay, where favorable conditions appear to drive the local growth of this organism, especially in warm late-summer months. Furthermore, V. coralliilyticus represented a large fraction of total Vibrio populations in WCSH larval rearing tanks, implying the persistence of this pathogen under hatchery conditions.

### Local growth of V. coralliilyticus in Netarts Bay.

**FIG 5:**
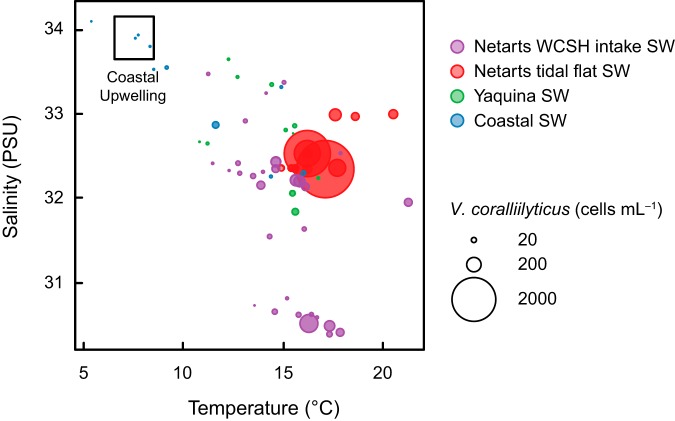
Concentrations V. coralliilyticus in all seawater samples plotted over the temperature and salinity of seawater. The upper-left-hand box contains samples collected from 200 m at station (Stn.) CE0405, thus reflecting the approximate temperature and salinity expected for upwelling source water. PSU, practical salinity units.

There is an economic incentive to understand the environmental drivers of V. coralliilyticus abundances in Netarts Bay, where a local shellfish hatchery (WCSH) is vulnerable to aquatic pathogens in seawater resources. A study by Elston et al. (16) suggested that V. coralliilyticus abundances in Netarts Bay might be related to oceanographic conditions, postulating that the upwelling of deep waters delivers high concentrations of this pathogen into the bay, and that subsequent relaxation and warming could fuel explosive growth. We did not find evidence to support this hypothesis. Concentrations of both Vibrio spp. and V. coralliilyticus decreased with depth at coastal stations (Fig. 3), indicating that upwelling conditions would supply lower V. coralliilyticus concentrations to the bay than downwelling conditions. Additionally, high concentrations of Vibrio spp. and V. coralliilyticus in Netarts WCSH intake water were not associated with physical or chemical characteristics of the cold, salty, nutrient-rich upwelling source water (Fig. 5); in fact, the highest concentrations in this time series occurred during a period of low salinity and warm temperatures (Fig. S6). The lack of evidence for a positive relationship between V. coralliilyticus abundance and coastal upwelling in the bay suggests that the threat of Vibrio pathogens to bivalves may be temporally uncoupled from ocean acidification stress in this environment.

We postulate that occurrences of elevated V. coralliilyticus abundance in Netarts Bay are not due to the advection of populations from coastal waters, but rather that favorable estuarine conditions promote the local growth of this organism. In this study, the highest concentrations of planktonic V. coralliilyticus were observed in tidal flat samples, especially in seawater sampled directly above shallow seagrass/macroalgal beds (Fig. 1). Our tidal flat samples were collected during very low-tide events, during which the seagrass/macroalgal bed stations were partially isolated and only connected to the larger bay by small tidal channels. These shallow tidal flat pools may act as environmental incubators, where higher residence times minimize the dilution of estuarine V. coralliilyticus populations with coastal seawater. It is also likely that these shallow stagnant waters are heated more efficiently than deeper channels, promoting the growth of total Vibrio spp. and V. coralliilyticus (24). Furthermore, concentrations of V. coralliilyticus in Netarts WCSH intake pipe samples were higher during low tide than during high tide, suggesting that the tidal input of nearshore oceanic waters dilutes local Vibrio populations in Netarts Bay. This contrasts with trends for total heterotrophic bacteria, whose concentrations were not correlated with tidal conditions, and it suggests a unique ecology of V. coralliilyticus.

Sediment reservoirs may also seed Vibrio populations into Netarts Bay seawater. Median concentrations of Vibrio spp. were an order of magnitude higher in tidal flat sediments than in tidal flat seawater and two orders of magnitude higher than in coastal seawater (Fig. 3), consistent with previous reports of sediments as reservoirs for Vibrio spp. in estuaries (28–30). The overall Vibrio community structure in Netarts Bay seawater was similar to that of the Netarts Bay sediment (Fig. 2), suggesting an interaction between these two reservoirs, which may help explain the high concentrations of Vibrio spp. in shallow tidal flat pools. However, the tidal flat sediment Vibrio communities contained a smaller relative proportion of V. coralliilyticus than the overlying seawater, though this difference is not statistically significant (Fig. 3). It is possible that V. coralliilyticus is supplied from alternate estuarine sources, such as seagrass or macroalgae (31). The advection of coastal seawater may also transport V. coralliilyticus into the bay, but this would require high local growth rates to account for the elevated abundance in Netarts seawater.

The abundances of total Vibrio spp. and V. coralliilyticus increased throughout the summer in Netarts Bay. This suggests a seasonality of Vibrio abundance, possibly due to elevated temperatures driving increased growth rates in late-summer months. Indeed, Vibrio species concentrations were positively related to temperature in Netarts WCSH intake seawater samples, which agrees with numerous observations of correlations between Vibrio species concentrations and temperature (32, 33) (Table 2). While V. coralliilyticus concentrations were not significantly related to temperature, they were correlated with the day of the year, with the highest concentrations observed in late summer months when temperatures were highest. The lack of a direct relationship between V. coralliilyticus concentrations and temperature may be due to the strong daily temperature fluctuations in Netarts Bay, arising from tidal and diel processes, which mask the seasonal signal in discrete measurements. Elevated seawater temperatures have been linked to global increases in Vibrio species concentrations and to incidences of pathogenic Vibrio infections worldwide (34, 35); thus, Vibrio species concentrations in Netarts Bay could be expected to increase in future warming oceans. It should also be noted that our sampling year (2015) was characterized by anomalously high sea surface temperatures (∼2 to 3°C above climatological averages) in the Northeast Pacific (36), which may have further increased Vibrio growth rates and/or shifted community interactions (e.g., 37).

One challenge in inferring environmental controls from this data set is the high degree of small-scale variability in Vibrio abundance. The coefficient of variation among biological replicates was an order of magnitude larger for concentrations of Vibrio spp. and V. coralliilyticus than for concentrations of total heterotrophic bacteria (Table S3). This high variability likely reflects the stochastic collection of Vibrio spp. associated with large suspended particles. Metabolic flexibility and the ability to produce extracellular enzymes, including chitinase (38), allow Vibrio spp. to grow on a wide range of substrates; Vibrio spp. have been observed to be associated with zooplankton (39), phytoplankton (40), and marine detritus (41). The fraction of particle-associated Vibrio spp. can be substantial, with concentrations of plankton-associated Vibrio spp. (per gram of biomass) several orders of magnitude higher than concentrations of free-living Vibrio spp. (per milliliter of seawater) (42). Since we filtered seawater onto 0.2-μm-pore-size filters without a prefiltration step, our samples reflect both the free-living and particle-associated fractions. Further studies are needed to determine whether V. coralliilyticus is consistently associated with specific living (e.g., a planktonic or benthic organism) or detrital reservoirs.

### Hatchery conditions allow the growth or persistence of V. coralliilyticus.

Vibriosis is a serious disease for aquaculture systems, including larval oyster hatcheries (43, 44), and it has been argued that V. coralliilyticus infections may have contributed to severe mortality events observed in hatcheries located on the northwest coast of the United States over the past 2 decades (16). Hatchery outbreaks could theoretically result from high concentrations of Vibrio pathogens in seawater resources; alternatively, hatcheries could become contaminated with toxigenic Vibrio spp. and/or environmental conditions could trigger increased virulence or larval susceptibility (45, 46). Here, we report lower total concentrations of Vibrio spp. in WCSH larval tanks than in Netarts Bay but a shift in the Vibrio community structure, with V. coralliilyticus representing a 30-fold larger fraction of total Vibrio spp. in tanks relative to intake seawater (Fig. 3). Numerous previous studies have observed high concentrations of pathogenic Vibrio spp. within aquaculture tank water and shellfish tissue, with shellfish tissue often being connected with host disease (12, 47, 48). It should be noted that in our study, the absolute concentrations of V. coralliilyticus in larval tanks were well below those required to induce mortality under laboratory conditions (27), and no samples were collected during severe mortality events (A. Barton, personal communication). However, the striking dominance of V. coralliilyticus over other Vibrio species in larval rearing tanks compared to the Vibrio community structure in inflowing seawater implies that this pathogen is particularly successful under hatchery conditions or resistant to hatchery disinfection methods and/or implicates contamination within the hatchery.

Following the initial seed stock collapses and the work of Elston et al. (16), hatchery personnel across the industry undertook extensive measures to reduce Vibrio concentrations in hatchery waters (18). During summer 2015, these practices included sand filtering intake seawater, bubbling seawater with ozone prior to larval inoculation, disinfecting tanks between larval batches (every 2 to 3 days), and using sterile techniques when handling phytoplankton cultures (see Barton et al. [18] for detailed information on hatchery setup and disinfection practices). The significantly lower overall abundance of Vibrio spp. in WCSH larval tank water than in bay waters suggests that these measures have been generally effective at total Vibrio removal. However, the persistence of V. coralliilyticus at near, or even slightly enriched above, bay water levels could suggest that the cleaning measures are less effective at removing this pathogen.

It is also possible that the higher relative proportion of V. coralliilyticus in hatchery tanks is due to contamination within the hatchery. Previous studies have documented substantial levels of Vibrio spp. in phytoplankton cultures, oyster broodstocks, thiosulfate, and air within hatcheries using similar disinfection methods (16, 49). Here, we observed moderate V. coralliilyticus concentrations in larval tanks containing phytoplankton and several size classes of larvae, tanks containing fertilized eggs prior to the addition of food, and in tanks containing sand-filtered, heated seawater prior to the addition of any larvae or phytoplankton (Table 3). Thus, it is unlikely that the observed V. coralliilyticus bacteria were introduced to larval tanks solely through phytoplankton or broodstock contamination.

**TABLE 3:** Summary of conditions of 19 larval tank samples collected from WCSH during summer 2015

Tank ID^*a*^	Date	Larval stage or length	Food	Time in tank prior to sampling	Bacterial cells · ml^−1^	Vibrio cells · ml^−1^	V. coralliilyticus/total Vibrio spp. (%)	V. coralliilyticus cells · ml^−1^
2	5 May	D-hinge	Yes	24 h	3.7 × 10^6^	2	F^*b*^	F
4	12 July	None	No	<1 day	1.1 × 10^6^	1,012	22	226
5	12 July	None	No	<1 day	1.1 × 10^6^	775	17	132
15	15 July	D-hinge	Yes	3 days	1.5 × 10^6^	406	F	F
16	15 July	D-hinge	Yes	3 days	9.9 × 10^5^	7	F	F
9 T = 0 h	27 July	Eggs	No	2 h	6.2 × 10^5^	110	13	15
9 T = 24 h	28 July	Eggs	Yes	1 day	7.4 × 10^5^	1,204	19	228
13 T = 0 h^*c*^	28 July	100–120 μm	Yes	6 h	1.2 × 10^6^	11 (0.2)	F	F
13 T = 40 h^*c*^	30 July	100–120 μm	Yes	2 days	1.6 × 10^6^	7 (4)	0/F	0/F
10^*c*^	12 August	90–110 μm	Yes	3 days	6.9 × 10^5^	77 (9)	F	F
7^*c*^	12 August	90–110 μm	Yes	3 days	6.8 × 10^5^	29 (39)	F	F
9 T = 0 h	8 September	Eggs	No	2 h	1.6 × 10^6^	294	37	110
9 T = 19 h	9 September	Eggs	No	1 day	1.4 × 10^6^	611	27	167
3	9 September	D-hinge	Yes	3 days	1.6 × 10^6^	403	72	290
9 T = 4 h	10 September	Eggs	No	2 days	8.2 × 10^5^	322	42	135

a ID, identification.

b F, failed PCR.

c Replicate samples. Concentrations for these samples represent averages, with standard deviations presented in parentheses.

Finally, it is possible that the observed V. coralliilyticus dominance in hatchery samples reflects high growth rates of this pathogen within the hatchery. Treating inflowing seawater with sand filtration and ozone likely reduced the concentration of total Vibrio spp., but subsequent heating to ∼25°C may have favored the growth of V. coralliilyticus. In fact, a previous study demonstrated that elevating seawater temperature can increase the abundance of V. coralliilyticus relative to other Vibrio species (24). While our study demonstrates a clear shift in Vibrio community structure from the Netarts WCSH intake seawater to the WCSH larval tank water, the underlying mechanisms for this shift remain speculative.

### Implications for virulence and larval disease.

Using abundances of pathogens, such as V. coralliilyticus, to track and predict toxicity is a major challenge due to the frequent nonlinearity between pathogen abundance, toxin production, and host mortality (see, e.g., reference 50). This is especially problematic for V. coralliilyticus, for which the mechanisms for virulence are not fully understood. While the extracellular zinc-metalloprotease VcpA can cause disease (51–53), recent studies have demonstrated that this metalloprotease is not required for pathogenesis in hosts, including oyster larvae (54, 55). Sequencing the genomes of V. coralliilyticus isolates has revealed a diverse repertoire of potential virulence factors (54, 55) which may function independently or in concert to induce pathogenicity (56).

Furthermore, the pathogenicity of V. coralliilyticus in seawater and hatcheries is likely regulated by environmental cues and community interactions. Seawater temperature is positively related to V. coralliilyticus growth and pathogenicity (24, 51), which could contribute to the success of V. coralliilyticus in bivalve hatcheries, where seawater is typically heated to improve larval growth. Ocean acidification conditions may also facilitate Vibrio species infections in bivalves (45), which could have severe consequences for the shellfish industry in the Northeast Pacific coastal upwelling zone, which is already threatened by the intrusion of acidified upwelled waters and bracing for future changes to carbonate chemistry (17, 18). Additionally, V. coralliilyticus pathogenicity may be affected by community interactions with other bacterial species, including Vibrio spp. (24, 56, 57), for instance, via the density-dependent production of quorum sensing molecules regulating virulence factors (58) and/or synergistic infections with other pathogens (59). In the current study, WCSH larval tank samples included dominant phylotypes classified as V. coralliilyticus, as well as the V. tubiashii pathogen and putatively benign Vibrio species, including V. penaeicida, V. lentos, and Aliivibrio fischeri (Fig. S3 and S4). This diverse community may collaborate to facilitate V. coralliilyticus infections. Furthermore, our study focused on oyster-pathogenic Vibrio spp., but our observations of V. cholerae sequences in seawater from Yaquina Bay suggest that coastal Oregon environments may need to be monitored for human-pathogenic Vibrio spp. in the future.

While more work is needed to elucidate how physical, chemical, and biological cues regulate V. coralliilyticus pathogenicity in hatcheries and coastal ecosystems, this study provides a critical first step toward understanding the ecology of this pathogen in temperate estuary systems. Netarts Bay appears to be a favorable environment for V. coralliilyticus, with sediment reservoirs likely seeding Vibrio populations and with shallow tidal pools allowing for local growth of this pathogen. Our findings of higher V. coralliilyticus abundances at low tide and in late summer could be used to inform practices in local hatcheries, where the threat of Vibrio pathogens is expected to worsen as temperatures increase in the coming decades. Furthermore, the stark community shift in Vibrio populations from Netarts WCSH intake seawater to larval tank water suggests that V. coralliilyticus infections could be a concern even when concentrations in the bay are low. Future work exploring the mechanisms and environmental controls of the toxicity of V. coralliilyticus are needed in order to evaluate the risks imposed by this pathogen.

## MATERIALS AND METHODS

### Sample collection.

Biological (DNA, flow cytometry, and chlorophyll *a*) and chemical (temperature, salinity, carbonate system parameters, nitrate plus nitrite, and phosphate) samples were collected from Oregon estuaries and coastal seawater during summer 2015 (Table 1). Estuary samples were collected from Netarts Bay, a shallow, tidally dominated bay located on the northern Oregon coast, and from Yaquina Bay, a drowned river estuary in central Oregon. Estuarine sampling was designed to encompass a range of tidal heights, times of day, offshore wind stress, and local chemistry. Additionally, coastal seawater samples were collected on research cruises in October 2014 and September 2015.

In Netarts Bay, samples were collected from tidal flat stations and from inflowing seawater and larval rearing tanks at the Whiskey Creek Shellfish Hatchery (WCSH), located on the eastern edge of the bay (Fig. 1). Seawater is continuously pumped from the bay into WCSH through a pipe located ∼0.5 m above a seagrass bed where the water depth is on average ∼2 m. Seawater samples were collected from the WCSH outflow of this pipe (∼5 s pipe residence time), which also flows through an analytical system measuring water temperature (T), salinity (S), the partial pressure of carbon dioxide (P_CO2_), and total carbon dioxide (TCO_2_). These samples (“Netarts WCSH intake SW”) thus represent natural seawater from Netarts Bay. Additionally, water samples were collected from WCSH larval rearing tanks (Table 3). These tanks are filled once every ∼3 days with seawater which has been collected from the inflow pipe and then sand filtered, UV treated, and heated to 25°C; subsequently, tanks are inoculated with larvae or sperm and eggs. Thus, larval tank samples reflect Netarts Bay seawater that has been chemically and biologically altered. On three occasions during low tide, seawater and sediment samples were also collected from stations in the Netarts Bay tidal flats. Sampling locations included tidal channels, isolated seawater pools, seagrass beds, mudflats, and sand flats (Fig. 1).

In Yaquina Bay, samples were collected from the Oregon State University pumphouse dock, located ∼2.5 km from the mouth of the Yaquina river (44.62°N, −124.04°W). A handheld Niskin sampling bottle was used to collect samples from approximately 1 m above the bottom, where a YSI 6600 series sonde equipped with T and S sensors was moored. The average sea level at this station was approximately 3 m.

Coastal seawater samples were collected at continental shelf and slope stations on and near the Newport Hydroline on cruises of opportunity in September 2015 (R/V Elahka) and October 2015 (R/V Oceanus) (Table 1). On both cruises, samples were collected with Niskin sampling bottles attached to a conductivity, temperature, depth (CTD) rosette. Sampling depths targeted the surface mixed layer, bottom water, and the oxycline.

### DNA preservation, extraction, amplification, and sequencing.

Seawater and tank water samples used for subsequent DNA extraction were sampled in duplicate into triple-rinsed 1- or 2-liter dark polycarbonate bottles, and between 300 and 1,700 ml was immediately filtered onto 25- or 47-mm diameter 0.2-μm-pore-size polyethersulfone Supor filters (Pall Corporation) using a peristaltic pump. Filters were placed into microcentrifuge tubes, flash-frozen, and transported in liquid nitrogen to Oregon State University, where they were stored at −80°C until analysis. DNA was extracted from filters using the DNeasy plant minikit (Qiagen), with a modified protocol to include additional steps for cell disruption through flash-freezing, bead beating with 200 μl of mixed 0.1-mm and 0.5-mm glass beads (Biospec Products), and proteinase K treatment. For Netarts Bay sediment samples, acid-washed plastic syringe corers were used to collect duplicate samples of the top 1 mm of sediment from each station. Sediment samples were transferred into Whirl-Pak bags (Nasco) and transported on dry ice to Oregon State University, where they were stored at −80°C. DNA was extracted from 0.25 g of sediment using the DNeasy PowerSoil DNA isolation kit (Qiagen), according to the manufacturer's instructions for wet soil samples. DNA extracts were quantified with the Quant-iT PicoGreen double-stranded DNA (dsDNA) assay kit (Invitrogen) using a MicroMax 384 plate reading fluorometer and stored at −20°C or −80°C.

The community composition of Vibrio spp. was analyzed via 16S rRNA gene sequencing. Vibrionaceae-specific primers developed by Yong et al. (60) and Thompson et al. (23) were used to amplify a 491-bp product in the V2–V4 regions of Vibrionaceae 16S rRNA genes. This primer set targets 68% of Vibrionaceae and 86% of Vibrio species sequences in the Silva database (SILVA TestPrime 1.0; https://www.arb-silva.de/search/testprime/, tested 3 January 2018 and allowing for no mismatches). An alignment of the primer set with 133 publically available Vibrionaceae 16S rRNA gene sequences is presented in Fig. S8.

DNA was amplified using a two-stage targeted amplicon sequencing approach (61, 62). In the first stage, the PCR was performed using primers that contained the gene-specific regions VF169 (60) and 680R (23) and common sequence tags, as described previously (63). These reactions were performed using DNAEngine (Bio-Rad) thermocyclers and 15-μl reaction mixture volumes consisting of 1× HotStarTaq *Plus* master mix (Qiagen), 1 μl DNA extract (diluted 1:10 in PCR-clean water), and 0.2 μM forward and reverse primers. The reaction mixtures were cycled at 95°C for 2 min, followed by 35 cycles of 95°C for 15 s, 53°C for 30 s, and 72°C for 30 s, with a final 72°C extension for 10 min. For each sample, PCRs were run in triplicate, visualized by gel electrophoresis, pooled, and quantified as described above. Samples were only sequenced if they had three successful PCRs, excluding PCR negative controls and filter blank samples, which were sequenced despite the absence of visual gel bands after amplification.

PCR amplicons were shipped on dry ice to the DNA Services Facility at the University of Illinois at Chicago for further processing. Here, a second PCR amplification was performed using Access Array barcode library primers (Fluidigm) containing common sequence linkers, unique barcodes (reverse primer only), and Illumina adapters. These reactions were performed in a 10-μl reaction volume using MyTaq HS 2× mastermix (Bioline) and were cycled at 95°C for 5 min, followed by 8 cycles of 95°C for 30 s, 60°C for 30 s, and 72°C for 30 s. PCR products were purified and normalized using SequalPrep plates (Life Technologies), quantified using a Quant-iT PicoGreen dsDNA assay kit (Thermo Fisher) with a GENios Pro fluorescence microplate reader (Tecan), and pooled using an epMotion5075 liquid handling workstation (Eppendorf). Pooled libraries were spiked with 15% PhiX and sequenced using MiSeq Standard version 3 2 × 300-bp paired-end sequencing. The sequencing reaction was initiated using the Fluidigm sequencing primers targeting the gene-specific primer and common sequence tag regions. Demultiplexing of reads was performed on the MiSeq instrument using Illumina BaseSpace. Sequencing was performed at the W. M. Keck Center for Comparative and Functional Genomics at the University of Illinois at Urbana-Champaign (UIUC).

### Bioinformatic analyses.

Sequence reads from Vibrio species 16S rRNA amplicons were demultiplexed using the Illumina MiSeq Reporter (MSR) version 2.5.1. Paired-end reads were merged via the make.contigs command in mothur (64) using a deltaq value of 20 as an additional quality control measure because of the relatively short (∼54-bp) overlapping region for forward and reverse reads. This option discards reads through the merging process when forward and reverse reads have different bases at the same position and the difference in quality score is <20. Merged reads were screened for quality using screen.seqs in mothur, retaining sequences between 480 and 540 bp in length with no ambiguities and or homopolymers of >8 bp. Singletons were removed, operational taxonomic units (OTUs) were clustered at 97% nucleotide sequence similarity, and a chimera check was performed against the Gold ChimeraSlayer reference database using USearch (65). Sequences were subsampled to 7,145 sequences per sample.

Vibrio species OTUs with >100 sequences in the rarefied data set were aligned against sequences from 134 cultured Vibrionaceae species, and a maximum likelihood phylogenetic tree was constructed using PhyML (66). Nonmetric multidimensional scaling (NMDS) analyses were performed using QIIME (67).

### Vibrio species and heterotrophic bacterial quantification.

Droplet digital PCR (ddPCR) was used to quantify total Vibrio species 16S rRNA gene copies using a Bio-Rad QX200 system. The ddPCR method produces copy number estimates that agree with quantitative PCR (qPCR)-based methods (68) but has several advantages, including lowered susceptibility to PCR inhibitors, absolute quantification without external standards, and greater precision and reproducibility (69). The reaction mixtures consisted of 10 μl EvaGreen PCR mastermix (Bio-Rad), 200 nM 567F and 680R primers (23), and ∼2 ng DNA, with a total reaction volume of 20 μl. Droplet generation, PCR, and scanning were conducted at the Oregon State University Center for Genome Research and Biocomputing, according to the manufacturer's instructions (Bio-Rad), but using an annealing temperature of 57°C. Data were analyzed using the QuantaSoft analysis software package. Filter blank samples (clean filters which underwent extraction and processing steps) were included on the run, and a detection limit was calculated as the average plus three standard deviations of the results from triplicate filter blanks. Vibrio species 16S rRNA gene copies were converted to cell concentrations using assumptions of 10% DNA extraction efficiency, 12 16S rRNA genes per genome, and monoploidy. V. coralliilyticus abundance estimates were calculated by multiplying the total Vibrio species concentrations by the fraction of V. coralliilyticus obtained from 16S rRNA gene sequence data (Fig. S2).

We also attempted to directly quantify the *vcpA* metalloprotease genes of V. coralliilyticus via ddPCR, using a previously published primer set (designed using V. coralliilyticus strain RE22 [70]). This assay was conducted using the same reagents and conditions as the Vibrio-specific 16S rRNA ddPCR assay, except that reaction mixtures contained 70 nmol *vcpA* (formerly named *vtpA*) forward and reverse primers and ∼25 ng DNA. We conducted *vcpA* ddPCR assays for a subset of samples (*n* = 88), but *vcpA* concentrations were all below or near our calculated detection limit despite the successful quantification of positive-control DNA from V. coralliilyticus strain RE22. Thus, no additional samples were analyzed, and these data are not presented in the manuscript.

Heterotrophic bacterial cell densities in seawater samples were measured using flow cytometry. Duplicate 3-ml subsamples were pipetted into cryovials and fixed with fresh paraformaldehyde at a final concentration of 1%. Fixed samples were inverted and incubated at room temperature in the dark for 10 min, flash-frozen, transported in liquid nitrogen to Oregon State University, and stored at −80°C. For analysis, samples were thawed on ice in the dark then spiked with Fluoresbrite 1-μm beads, stained with SYBR green I (71), and run on a Becton-Dickinson FACSCalibur flow cytometer with a 488-nm laser. Bacterial cells and beads were distinguished from other particulate matter by their side light scatter and green fluorescence.

### Ancillary data.

Discrete samples were preserved for chlorophyll *a* (Chl *a*), nitrate plus nitrite, and phosphate. For Chl *a*, 50 ml seawater was filtered onto 25-mm GF/F filters (Whatman), which were placed in snap-cap tubes, wrapped in foil, and flash-frozen. For nutrient samples, 25 ml seawater was frozen in high-density polyethylene bottles. Samples for Chl *a* and nutrients were transported in liquid nitrogen to OSU and stored at −80°C until analysis. Chl *a* was extracted in acetone at −20°C for 48 h and then analyzed with a Turner Model 10-AU fluorometer using the methods of Welschmeyer (72). Nutrient samples were thawed, filtered through 25-mm GF/F filters (Whatman), and analyzed via phosphomolybdic acid reduction for phosphate and cadmium reduction (73) for nitrate plus nitrite using a Technicon Auto Analyzer II.

At WCSH, the P_CO2_ and TCO_2_ of hatchery intake seawater were measured in real time using the Burke-O-Lator 3000 (see reference 18). Tidal heights were estimated for the Yaquina Bay and Netarts WCSH intake time points using the program XTide (available at http://www.flaterco.com/xtide/) using the South Beach, Yaquina Bay, OR and Netarts, Netarts Bay, OR sites, respectively. Daily wind stress was derived from winds observed at Newport, OR (http://damp.coas.oregonstate.edu/windstress/).

### Statistical analyses.

Concentrations of bacteria, Vibrio spp., and V. coralliilyticus were log-transformed prior to all regressions in order to improve model assumptions of normality and equal variance. One-way ANOVA with subsequent Tukey honestly significant difference (HSD) tests of multiple comparisons were performed using the program R (http://www.r-project.org/) to test for differences in percentages of V. coralliilyticus sequences and in log-transformed *Vibrio* species and V. coralliilyticus concentrations among sample types. One-way ANOVA and Tukey HSD tests were also used to test for differences in concentrations among days and stations. Indicator phylotypes were identified using an indicator species analysis (74) with the R package labdsv (75). Additionally, a one-way analysis of similarity (ANOSIM) was used to test for significant differences in Vibrionaceae community structure among sample types. The ANOSIM was performed using Bray-Curtis values without transformation via the PRIMER software version 7.0.

Four separate type II linear regression models were used to test for environmental predictors of V. coralliilyticus, Vibrio spp., total heterotrophic bacteria (volumetric concentrations), and the ratio of V. coralliilyticus to total heterotrophic bacteria in the Netarts WCSH intake seawater samples. Graphical analysis did not indicate strong colinearity between explanatory variables tested (temperature, salinity, daily wind stress, discrete tide, P_CO2_, nitrate plus nitrite, and phosphate). Initial models incorporated all explanatory variables, and then subsequent models were reduced to incorporate only variables with statistically significant predictive power (*P* < 0.05); summary statistics are reported from the reduced models. The relative contribution of each explanatory variable to the total *R*^2^ value of each model was calculated using the R package relaimpo (76). Analysis of residuals using the acf function in R indicated no problems with temporal autocorrelation.

### Accession number(s).

Raw sequence data are available from the NCBI under accession no. SRP118403.

## Supplementary Material

Supplemental material
