# Supplementary material for: Environmental Controls of Oyster-Pathogenic Vibrio spp. in Oregon Estuaries and a Shellfish Hatchery
Source: Appl Environ Microbiol. 2018 Apr 16;84(9):e02156-17. doi: 10.1128/AEM.02156-17 (PMC5930336; doi:10.1128/AEM.02156-17)
Supplement: Supplemental material [file supp_84_9_e02156-17__index.html]

Supplemental material 

# Environmental Controls of Oyster-Pathogenic Vibrio spp. in Oregon Estuaries and a Shellfish Hatchery

## Supplemental material

- Supplemental file 1 -

  Rarefaction curves for all samples sequenced in this study (Fig. S1); maximum likelihood phylogenetic tree depicting *Vibrio* 16S rRNA gene sequences from this study (Fig. S2); relative abundances of dominant *Vibrio* spp. operational taxonomic units across all DNA samples (Fig. S3); average percentage of total *Vibrio* spp. classified as putative pathogens from categories of DNA collected in 2014 and 2015 (Fig. S4); DNA-normalized concentrations of *V. coralliilyticus* and total *Vibrio* spp. (Fig. S5); concentrations of *Vibrio* spp. and *V. coralliilyticus* along with physicochemical parameters from seawater sampled from the Netarts WCSH inlet during summer 2015 (Fig. S6); *V. coralliilyticus* concentrations in seawater sampled from from Netarts tidal flat stations (Fig. S7); nucleotide alignment showing mismatches between the 16S rRNA sequences of 133 publicly available *Vibrionaceae* species and the binding sites of the *Vibrio*-specific primers used in this study (Fig. S8); range of physical, chemical and biological conditions of seawater samples over the summer 2015 sampling period for Netarts Bay and Yaquina Bay, and over the depth profile for coastal stations (Table S1); indicator phylotypes identified by an indicator species analysis (Table S2); average coefficient of variation for concentrations of total *Vibrio* spp., *V. coralliilyticus*, and total heterotrophic bacteria observed at different temporal and spatial scales in this study (Table S3).

  PDF, 8.4M
