# Supplementary material for: Environmental Controls of Oyster-Pathogenic Vibrio spp. in Oregon Estuaries and a Shellfish Hatchery
Source: Appl Environ Microbiol. 2018 Apr 16;84(9):e02156-17. doi: 10.1128/AEM.02156-17 (PMC5930336; doi:10.1128/AEM.02156-17)
Supplement: Supplemental material [file AEM.02156-17_zam009188475s1.pdf]

SUPPLEMENTARY INFORMATION

Environmental Controls of Oyster-pathogenic *Vibrio spp.* in Oregon Estuaries and a Shellfish Hatchery

Mary R. Gradoville, Byron C. Crump, Claudia C. Häse, and Angelicque E. White

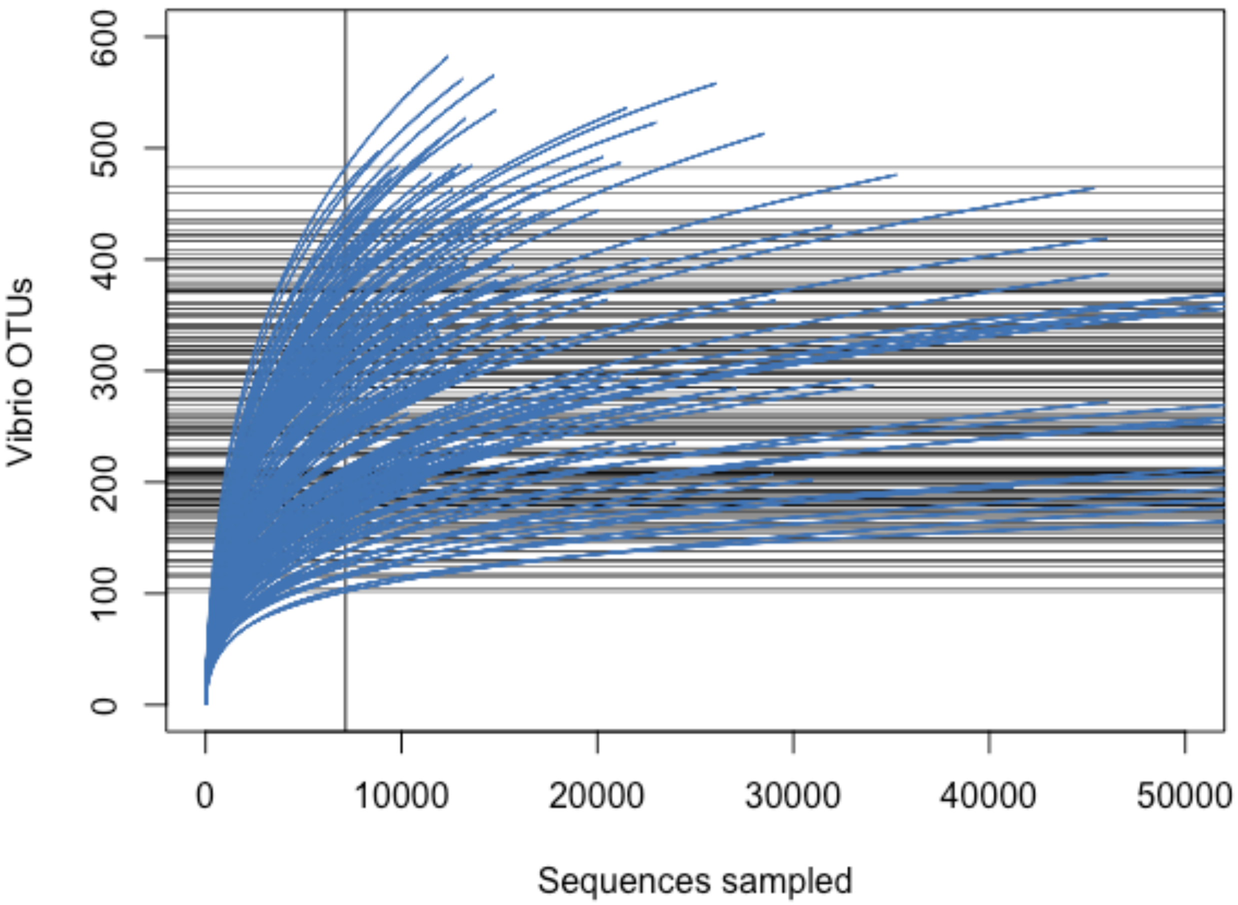

Figure S1: Rarefaction curves for all samples sequenced in this study. Curves are based on *Vibrio* spp. OTUs clustered at 97% nucleotide identity, and were produced using the vegan rarecurve function (http://CRAN.R- project.org/package=vegan). All samples were subsampled to 7145 sequences (vertical line).

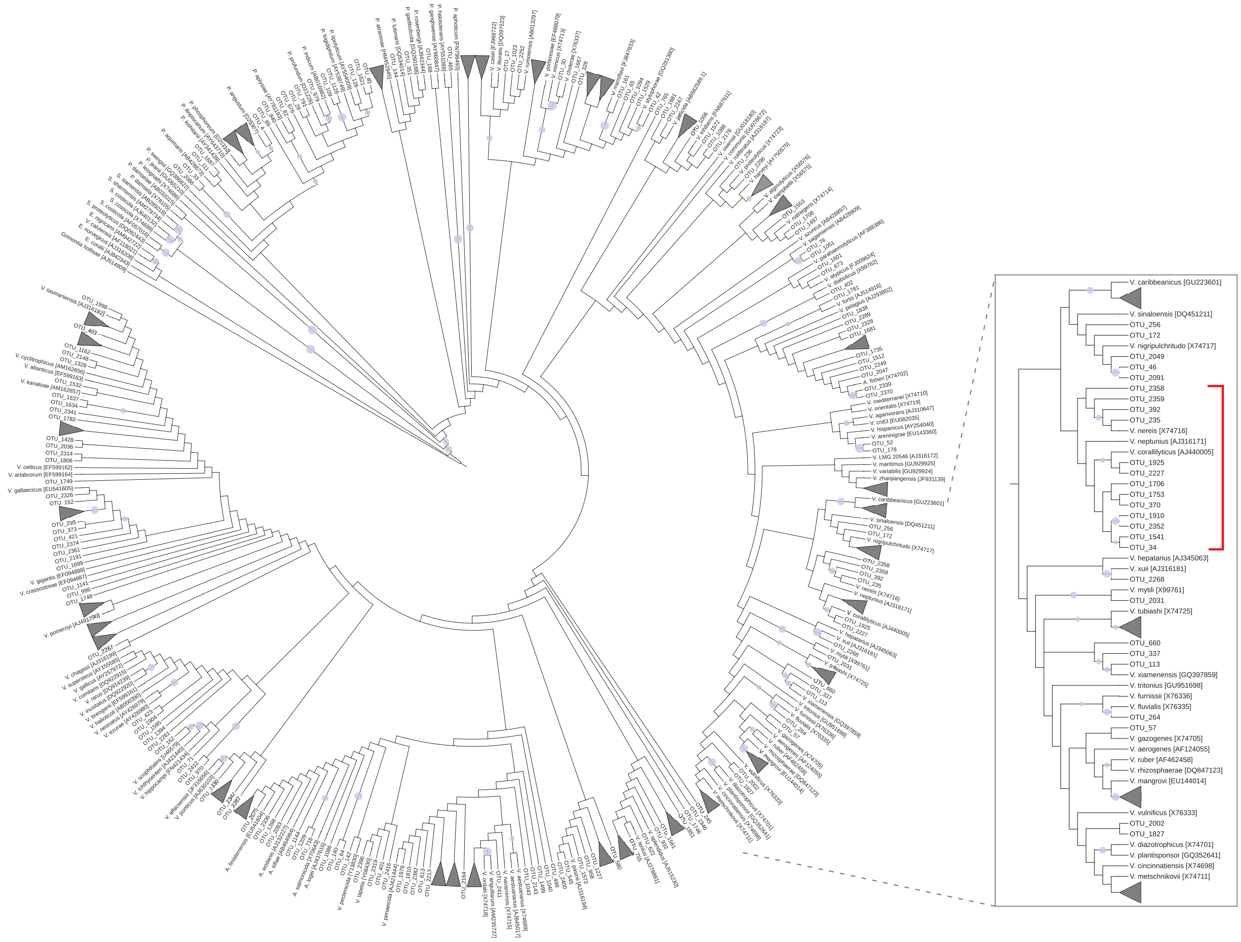

Figure S2: Maximum likelihood phylogenetic tree depicting Vibrionaceae 16S rRNA gene sequences from this study. A representative sequence from OTUs containing >100 sequences in the rarefied dataset (>98% of total rarefied sequences) and sequences from 134 Vibrionaceae isolates are displayed on the tree. The right panel shows a section of the tree which includes the 13 OTUs classified as *V. coralliilyticus*. Bootstrap values (100 replicates) of >50% are represented with size-proportional violet circles. Nodes containing OTUs only and no reference sequences were collapsed. The tree was produced using the Interactive Tree of Life (http://itol.embl.de/).

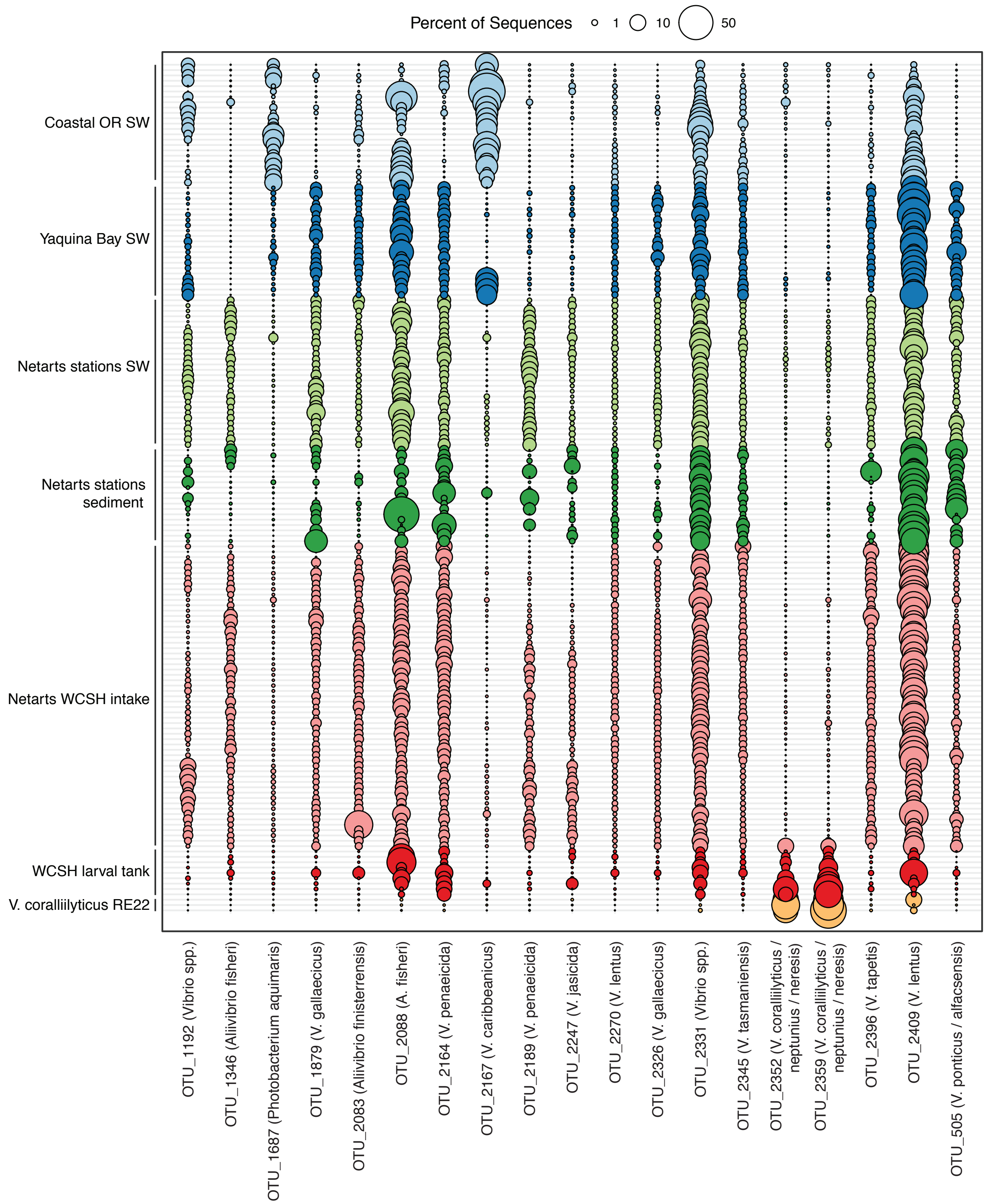

Figure S3: Relative abundances of dominant *Vibrio* spp. OTUs across all DNA samples. The 19 most abundant OTUs are depicted, representing >65% of total sequences from the rarefied

dataset. Note that OTUs identified as *V. coralliilyticus* also clustered with *V. neptunius* and *V. neresis*, and that OTUs identified as *V. ponticus* also clustered with *V. alfacsensis*.

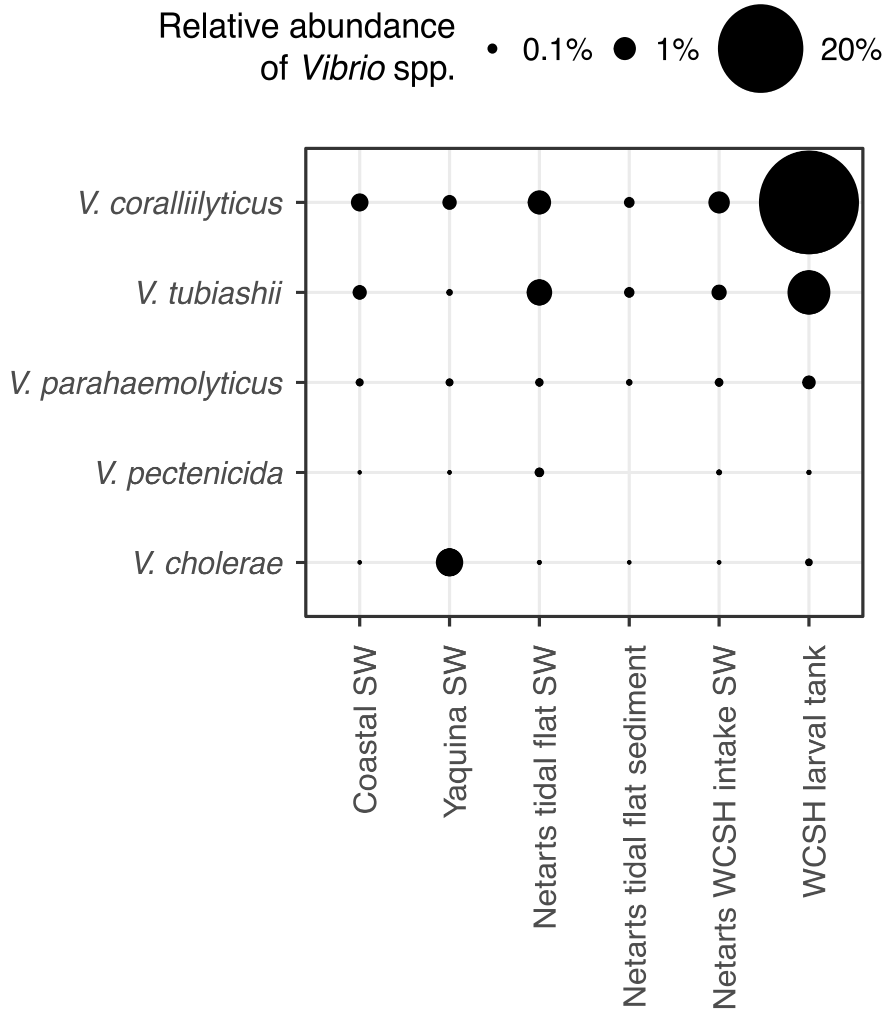

Figure S4: Average percentage of total *Vibrio* spp. classified as putative pathogens from the different types of DNA collected in 2014 and 2015.

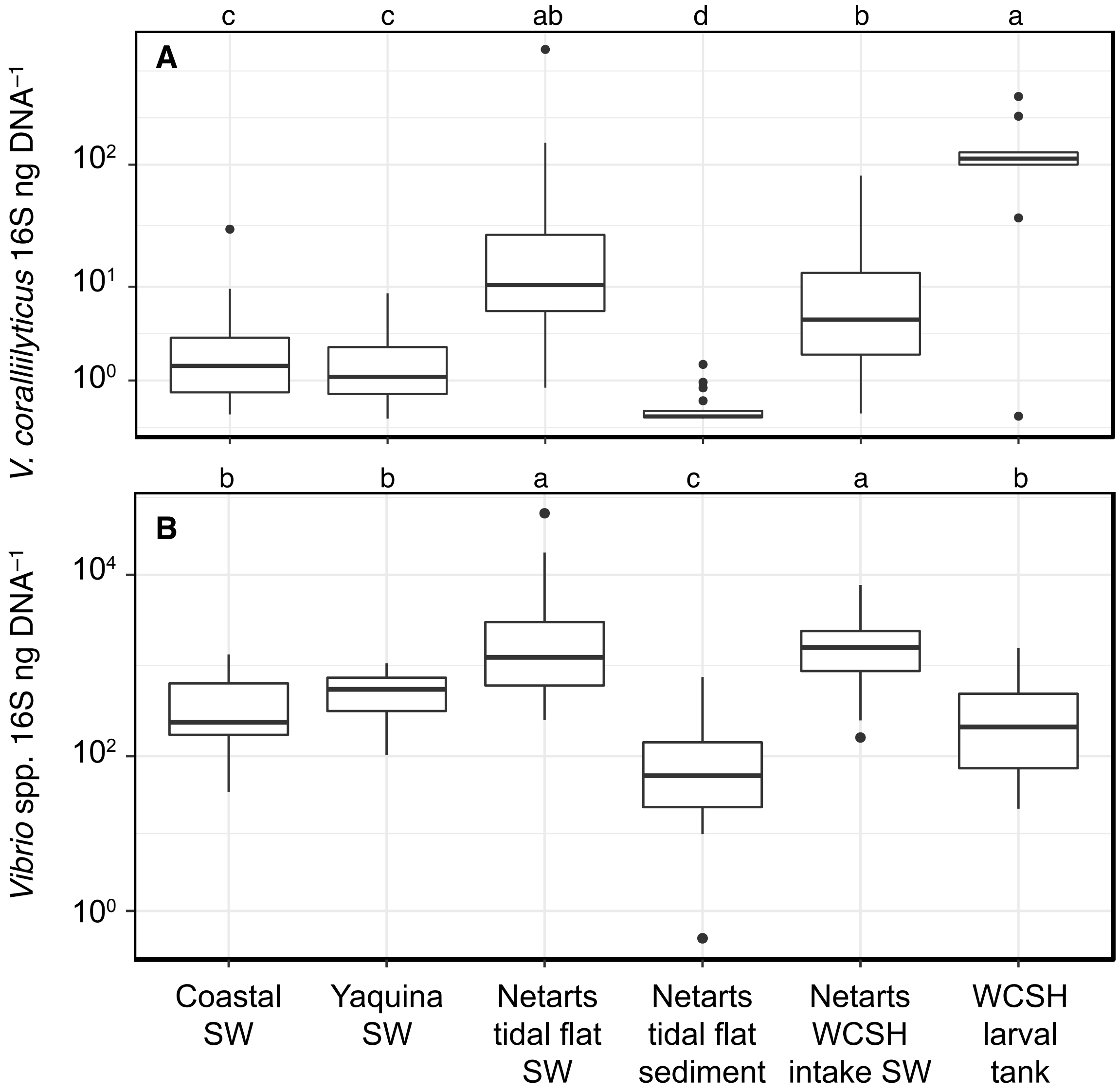

Figure S5: DNA-normalized concentrations of *V. coralliilyticus* (A) and total *Vibrio spp.* (B). Letters above each panel note statistical significance, where different letters signify significant differences in log-transformed means within a panel (Tukey HSD $p < 0.05$), and categories with the same letter are not statistically different from one another. Boxplots represent medians as thick horizontal lines, 25-75% quantiles as boxes, the smallest and largest values (at most 1.5 times the inter-quartile range) as whiskers, and outliers as dots.

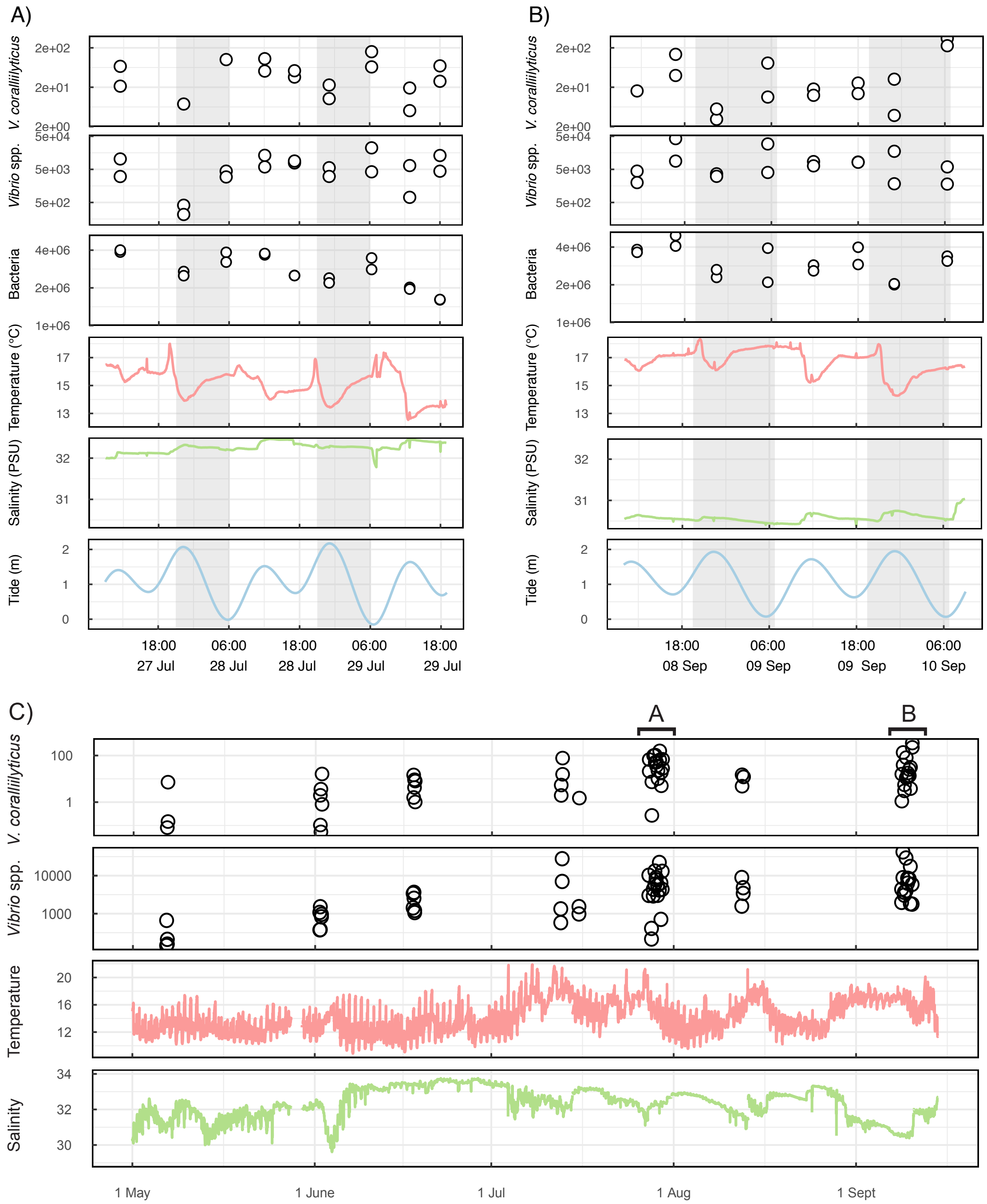

Figure S6: Concentrations of *Vibrio* spp. and *V. coralliilyticus* (cells mL^{-1}) along with physicochemical parameters (temperature, °C; salinity, PSU) from seawater sampled from the Netarts WCSH intake pipe during summer 2015. Concentrations of total heterotrophic bacteria

(cells mL^{-1}) and tidal height are presented for two intensive sampling periods (A, B) from the time series (C). Shaded columns in A and B represent night time.

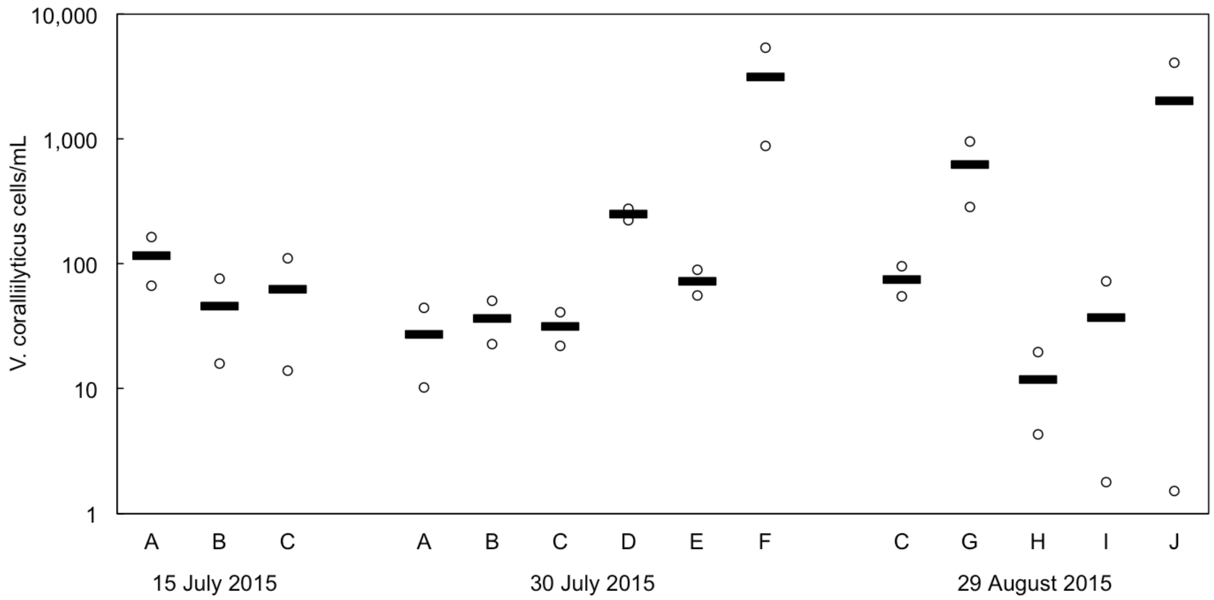

Figure S7: *V. coralliilyticus* concentrations in Netarts tidal flat seawater samples. Stations were sampled during low tide from ~07:00–09:00 on 15 July, 30 July, and 29 August 2015. Circles represent individual samples; dark bars represent averages. Letters represent sampling stations. See Fig. 1 for locations of sampling stations.

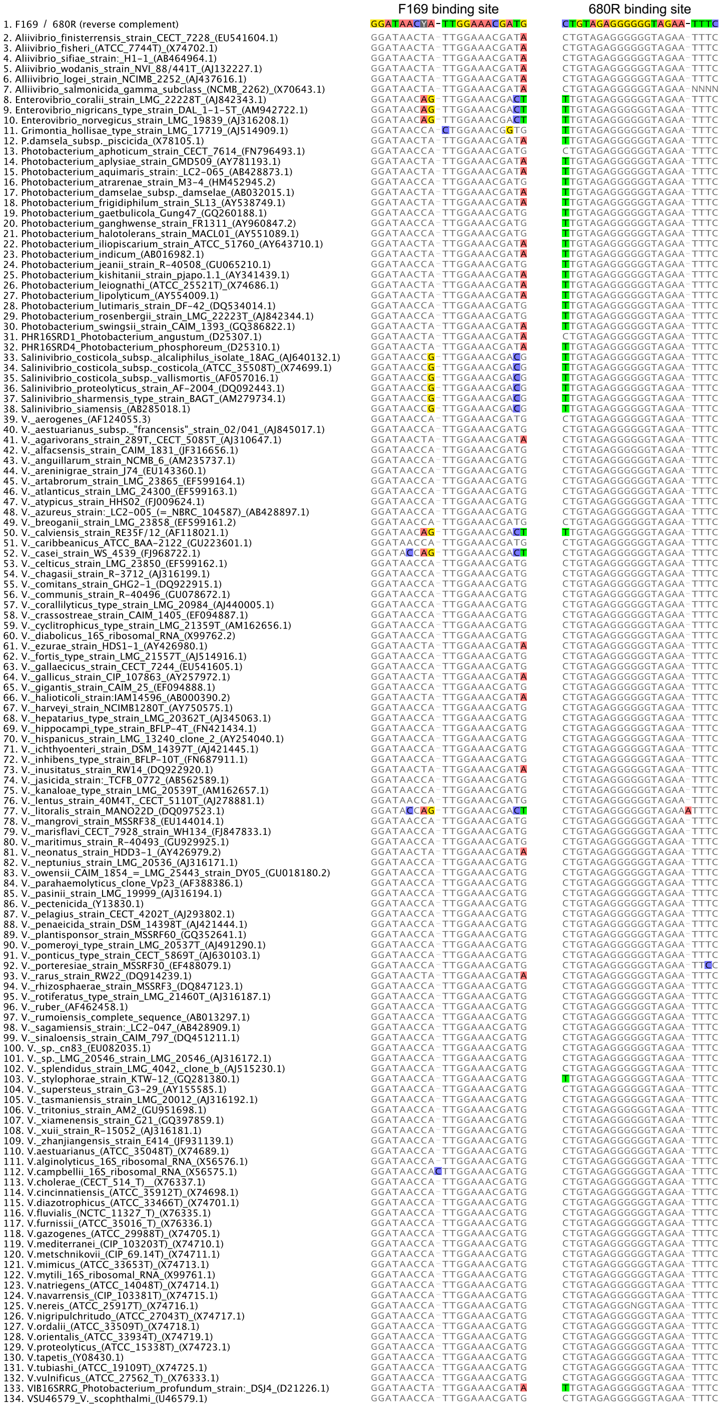

Figure S8: Nucleotide alignment showing mismatches between the 16S rRNA gene sequences of 133 publically available Vibrionaceae species and the binding sites of the *Vibrio*-specific sequencing primers used in this study (row 1).

Table S1: Ranges of physical, chemical and biological conditions of seawater sampled over the summer 2015 sampling period for Netarts Bay and Yaquina Bay, and over the depth profiles for coastal stations. ND indicates no data available.

**Location**	**Sampling time period**	**Temp (°C)**	**Sal (PSU)**	**N+N (μmol L^{-1})**	**PO_4 (μmol L^{-1})**	**P_{CO2} (μatm)**	**Chl *a* (μg L^{-1})**	**N wind (N m^{-2})**
Netarts WCSH intake SW	May – Sep 2015	11.2 – 21.3	30.4 – 33.5	0 – 4.2	0.5 – 4.7	245 – 940	1.77 – 10.2	–0.08 – 0.05
Netarts tidal flat SW	Jul – Aug 2015	14.9 – 20.5	ND	0 – 2.4	0.8 – 6.6	ND	2.2 – 20.7	–0.07 – 0.22
Yaquina SW	Jul – Sep 2015	10.8 – 16.8	31.8 – 33.7	0 – 6.4	0.7 – 1.7	ND	2.37 – 6.19	– 0.04 – 0.02
Coastal OR NH10	Oct 2014	8.5 – 14.9	33.3 – 33.5	0.2 – 26.1	0.5 – 2.3	301 – 887	1.0 – 7.1	0
Coastal OR CE0405	Oct 2014	5.4 – 16.0	32.3 – 34.1	0.2 – 40.6	0.3 – 3.0	331 – 944	0.01 – 1.46	–0.04
Coastal OR NH5	Sep 2015	9.2 – 11.6	32.9 – 33.6	0.1 – 8.2	0.7 – 1.8	ND	0.37 – 7.91	0.01
Coastal OR NH25	Sep 2015	7.7 – 14.4	32.3 – 33.9	0 – 12.3	0.4 – 2.2	ND	0.06 – 0.98	0.03

Table S2: Indicator phylotypes identificated by an indicator species analysis. Only statistically significant ($p < 0.05$) phylotypes are presented. OTUs clustering with *V. coralliilyticus* (Fig. S2) are shown in bold.

OTU	Group	Indicator value	*p*-value
**OTU_2359**	**WCSH Tank**	**0.954**	**0.001**
OTU_1781	WCSH Tank	0.882	0.001
**OTU_1541**	**WCSH Tank**	**0.878**	**0.001**
**OTU_2227**	**WCSH Tank**	**0.864**	**0.001**
OTU_1681	WCSH Tank	0.863	0.001
OTU_2112	WCSH Tank	0.847	0.001
**OTU_2358**	**WCSH Tank**	**0.820**	**0.001**
**OTU_2352**	**WCSH Tank**	**0.813**	**0.001**
**OTU_1925**	**WCSH Tank**	**0.719**	**0.001**
**OTU_34**	**WCSH Tank**	**0.657**	**0.001**
OTU_2289	WCSH Tank	0.655	0.001
OTU_1721	WCSH Tank	0.627	0.001
OTU_2230	WCSH Tank	0.612	0.001
OTU_2134	WCSH Tank	0.605	0.001
OTU_1738	WCSH Tank	0.586	0.001
OTU_1382	WCSH Tank	0.557	0.001
OTU_2178	WCSH Tank	0.553	0.001
OTU_1181	WCSH Tank	0.547	0.001
OTU_1497	WCSH Tank	0.547	0.001
OTU_2242	WCSH Tank	0.544	0.004
**OTU_1706**	**WCSH Tank**	**0.520**	**0.001**
OTU_1088	WCSH Tank	0.515	0.002
OTU_1805	WCSH Tank	0.506	0.001
OTU_115	WCSH Tank	0.489	0.001
OTU_1708	WCSH Tank	0.474	0.001
OTU_2303	WCSH Tank	0.472	0.002
**OTU_370**	**WCSH Tank**	**0.452**	**0.001**
OTU_2186	WCSH Tank	0.451	0.001
OTU_311	WCSH Tank	0.410	0.004
OTU_2377	WCSH Tank	0.376	0.005
OTU_1601	WCSH Tank	0.343	0.015
OTU_2153	WCSH Tank	0.340	0.014
OTU_2268	WCSH Tank	0.327	0.021
OTU_1006	WCSH Tank	0.327	0.006
**OTU_1910**	**WCSH Tank**	**0.306**	**0.001**
OTU_660	WCSH Tank	0.288	0.012
OTU_2002	WCSH Tank	0.287	0.003
OTU_765	WCSH Tank	0.273	0.005
OTU_1499	WCSH Tank	0.273	0.006
OTU_1040	WCSH Tank	0.272	0.031
OTU_2386	WCSH Tank	0.232	0.03
OTU_1687	Coastal SW	0.850	0.001
OTU_2101	Coastal SW	0.831	0.001

OTU_111	Coastal SW	0.768	0.001
OTU_2167	Coastal SW	0.747	0.001
OTU_444	Coastal SW	0.742	0.001
OTU_1170	Coastal SW	0.621	0.001
OTU_2263	Coastal SW	0.617	0.001
OTU_2285	Coastal SW	0.610	0.001
OTU_1993	Coastal SW	0.607	0.001
OTU_842	Coastal SW	0.603	0.001
OTU_1528	Coastal SW	0.590	0.001
OTU_4	Coastal SW	0.545	0.001
OTU_172	Coastal SW	0.520	0.011
OTU_1099	Coastal SW	0.508	0.001
OTU_2040	Coastal SW	0.467	0.001
OTU_2291	Coastal SW	0.467	0.001
OTU_1866	Coastal SW	0.453	0.001
OTU_32	Coastal SW	0.452	0.001
OTU_603	Coastal SW	0.448	0.002
OTU_1301	Coastal SW	0.446	0.002
OTU_2037	Coastal SW	0.425	0.004
OTU_76	Coastal SW	0.399	0.001
OTU_1784	Coastal SW	0.384	0.002
OTU_2121	Coastal SW	0.381	0.003
OTU_109	Coastal SW	0.374	0.003
OTU_2195	Coastal SW	0.343	0.006
OTU_1602	Coastal SW	0.335	0.003
OTU_2034	Coastal SW	0.326	0.008
OTU_83	Coastal SW	0.324	0.002
OTU_2402	Coastal SW	0.322	0.007
OTU_570	Coastal SW	0.317	0.002
OTU_1982	Coastal SW	0.309	0.005
OTU_1991	Coastal SW	0.295	0.02
OTU_2331	Coastal SW	0.291	0.003
OTU_1141	Coastal SW	0.283	0.007
OTU_964	Coastal SW	0.281	0.004
OTU_1442	Coastal SW	0.273	0.026
OTU_80	Coastal SW	0.270	0.001
OTU_1051	Coastal SW	0.267	0.006
OTU_1748	Coastal SW	0.263	0.009
OTU_2314	Coastal SW	0.256	0.046
OTU_1470	Coastal SW	0.249	0.017
OTU_492	Coastal SW	0.243	0.001
OTU_791	Coastal SW	0.242	0.032
OTU_1353	Coastal SW	0.227	0.042
OTU_1684	Coastal SW	0.215	0.003
OTU_613	Coastal SW	0.206	0.038
OTU_341	Coastal SW	0.202	0.024
OTU_1128	Coastal SW	0.202	0.015
OTU_264	Coastal SW	0.200	0.036
OTU_66	Coastal SW	0.191	0.005

OTU_140	Coastal SW	0.184	0.015
OTU_2296	Coastal SW	0.172	0.048
OTU_390	Coastal SW	0.162	0.013
OTU_126	Coastal SW	0.135	0.016
OTU_505	Netarts Stn. Sediment	0.485	0.001
OTU_200	Netarts Stn. Sediment	0.482	0.001
OTU_992	Netarts Stn. Sediment	0.444	0.001
OTU_829	Netarts Stn. Sediment	0.409	0.003
OTU_2243	Netarts Stn. Sediment	0.387	0.005
OTU_1881	Netarts Stn. Sediment	0.384	0.006
OTU_1995	Netarts Stn. Sediment	0.359	0.027
OTU_2129	Netarts Stn. Sediment	0.338	0.002
OTU_245	Netarts Stn. Sediment	0.337	0.016
OTU_2020	Netarts Stn. Sediment	0.327	0.015
OTU_2247	Netarts Stn. Sediment	0.326	0.015
OTU_2376	Netarts Stn. Sediment	0.311	0.008
OTU_2133	Netarts Stn. Sediment	0.311	0.003
OTU_2287	Netarts Stn. Sediment	0.303	0.017
OTU_236	Netarts Stn. Sediment	0.301	0.009
OTU_2373	Netarts Stn. Sediment	0.299	0.027
OTU_1746	Netarts Stn. Sediment	0.287	0.019
OTU_2279	Netarts Stn. Sediment	0.285	0.004
OTU_606	Netarts Stn. Sediment	0.283	0.002
OTU_1276	Netarts Stn. Sediment	0.282	0.019
OTU_1191	Netarts Stn. Sediment	0.270	0.006
OTU_979	Netarts Stn. Sediment	0.263	0.017
OTU_2164	Netarts Stn. Sediment	0.259	0.028
OTU_406	Netarts Stn. Sediment	0.252	0.016
OTU_2409	Netarts Stn. Sediment	0.249	0.001
OTU_2157	Netarts Stn. Sediment	0.248	0.019
OTU_1806	Netarts Stn. Sediment	0.243	0.02
OTU_1577	Netarts Stn. Sediment	0.243	0.017
OTU_815	Netarts Stn. Sediment	0.238	0.044
OTU_2067	Netarts Stn. Sediment	0.226	0.05
OTU_2149	Netarts Stn. Sediment	0.226	0.045
OTU_1886	Netarts Stn. SW	0.691	0.001
OTU_1930	Netarts Stn. SW	0.587	0.001
OTU_2382	Netarts Stn. SW	0.575	0.001
OTU_101	Netarts Stn. SW	0.573	0.001
OTU_1810	Netarts Stn. SW	0.558	0.001
OTU_681	Netarts Stn. SW	0.548	0.001
OTU_79	Netarts Stn. SW	0.548	0.001
OTU_207	Netarts Stn. SW	0.533	0.001
OTU_2189	Netarts Stn. SW	0.532	0.001
OTU_1359	Netarts Stn. SW	0.526	0.001
OTU_1976	Netarts Stn. SW	0.526	0.001
OTU_18	Netarts Stn. SW	0.518	0.001
OTU_2091	Netarts Stn. SW	0.512	0.001
OTU_117	Netarts Stn. SW	0.493	0.001

OTU_139	Netarts Stn. SW	0.459	0.001
OTU_186	Netarts Stn. SW	0.458	0.001
OTU_1913	Netarts Stn. SW	0.450	0.001
OTU_545	Netarts Stn. SW	0.446	0.001
OTU_75	Netarts Stn. SW	0.443	0.001
OTU_2400	Netarts Stn. SW	0.440	0.001
OTU_2049	Netarts Stn. SW	0.438	0.001
OTU_133	Netarts Stn. SW	0.436	0.001
OTU_142	Netarts Stn. SW	0.422	0.001
OTU_1023	Netarts Stn. SW	0.415	0.001
OTU_9	Netarts Stn. SW	0.410	0.001
OTU_52	Netarts Stn. SW	0.410	0.001
OTU_958	Netarts Stn. SW	0.410	0.001
OTU_401	Netarts Stn. SW	0.409	0.003
OTU_337	Netarts Stn. SW	0.407	0.001
OTU_1038	Netarts Stn. SW	0.407	0.001
OTU_17	Netarts Stn. SW	0.403	0.001
OTU_2213	Netarts Stn. SW	0.401	0.002
OTU_2214	Netarts Stn. SW	0.400	0.003
**OTU_1753**	**Netarts Stn. SW**	**0.397**	**0.001**
OTU_1008	Netarts Stn. SW	0.394	0.002
OTU_379	Netarts Stn. SW	0.393	0.001
OTU_329	Netarts Stn. SW	0.385	0.001
OTU_1663	Netarts Stn. SW	0.385	0.002
OTU_1346	Netarts Stn. SW	0.382	0.001
OTU_1227	Netarts Stn. SW	0.376	0.014
OTU_2299	Netarts Stn. SW	0.367	0.002
OTU_21	Netarts Stn. SW	0.366	0.033
OTU_2361	Netarts Stn. SW	0.354	0.02
OTU_113	Netarts Stn. SW	0.346	0.002
OTU_15	Netarts Stn. SW	0.346	0.004
OTU_957	Netarts Stn. SW	0.346	0.004
OTU_1529	Netarts Stn. SW	0.342	0.007
OTU_120	Netarts Stn. SW	0.331	0.002
OTU_1842	Netarts Stn. SW	0.328	0.001
OTU_43	Netarts Stn. SW	0.327	0.001
OTU_1140	Netarts Stn. SW	0.327	0.002
OTU_2094	Netarts Stn. SW	0.324	0.006
OTU_1958	Netarts Stn. SW	0.322	0.004
OTU_2261	Netarts Stn. SW	0.313	0.007
OTU_840	Netarts Stn. SW	0.313	0.017
OTU_1573	Netarts Stn. SW	0.310	0.005
OTU_1879	Netarts Stn. SW	0.305	0.016
OTU_12	Netarts Stn. SW	0.299	0.006
OTU_1829	Netarts Stn. SW	0.298	0.017
OTU_25	Netarts Stn. SW	0.294	0.005
OTU_178	Netarts Stn. SW	0.291	0.003
OTU_55	Netarts Stn. SW	0.284	0.015
OTU_2288	Netarts Stn. SW	0.281	0.017

OTU_1094	Netarts Stn. SW	0.267	0.017
OTU_82	Netarts Stn. SW	0.266	0.015
OTU_2087	Netarts Stn. SW	0.260	0.014
OTU_50	Netarts Stn. SW	0.251	0.025
OTU_47	Netarts Stn. SW	0.251	0.003
OTU_2374	Netarts Stn. SW	0.248	0.011
OTU_421	Netarts Stn. SW	0.244	0.018
OTU_498	Netarts Stn. SW	0.242	0.03
OTU_836	Netarts Stn. SW	0.241	0.016
OTU_69	Netarts Stn. SW	0.241	0.005
OTU_1454	Netarts Stn. SW	0.237	0.014
OTU_1215	Netarts Stn. SW	0.234	0.012
OTU_144	Netarts Stn. SW	0.234	0.037
OTU_1260	Netarts Stn. SW	0.230	0.023
OTU_2355	Netarts Stn. SW	0.227	0.032
OTU_57	Netarts Stn. SW	0.227	0.005
OTU_1340	Netarts Stn. SW	0.218	0.025
OTU_1375	Netarts Stn. SW	0.211	0.012
OTU_143	Netarts Stn. SW	0.209	0.026
OTU_566	Netarts Stn. SW	0.194	0.024
OTU_2075	Netarts WCSH inflow	0.558	0.004
OTU_1978	Netarts WCSH inflow	0.520	0.001
OTU_2047	Netarts WCSH inflow	0.467	0.002
OTU_2354	Netarts WCSH inflow	0.455	0.001
OTU_2	Netarts WCSH inflow	0.440	0.001
OTU_51	Netarts WCSH inflow	0.421	0.003
OTU_1485	Netarts WCSH inflow	0.416	0.001
OTU_2083	Netarts WCSH inflow	0.400	0.025
OTU_1670	Netarts WCSH inflow	0.386	0.001
OTU_1090	Netarts WCSH inflow	0.382	0.003
OTU_2342	Netarts WCSH inflow	0.375	0.001
OTU_2105	Netarts WCSH inflow	0.372	0.012
OTU_1379	Netarts WCSH inflow	0.370	0.01
OTU_2328	Netarts WCSH inflow	0.369	0.001
OTU_2348	Netarts WCSH inflow	0.364	0.004
OTU_2393	Netarts WCSH inflow	0.354	0.006
OTU_1838	Netarts WCSH inflow	0.353	0.003
OTU_617	Netarts WCSH inflow	0.345	0.004
OTU_2336	Netarts WCSH inflow	0.343	0.008
OTU_40	Netarts WCSH inflow	0.341	0.002
OTU_2396	Netarts WCSH inflow	0.338	0.004
OTU_2387	Netarts WCSH inflow	0.333	0.029
OTU_2252	Netarts WCSH inflow	0.332	0.001
OTU_1272	Netarts WCSH inflow	0.325	0.007
OTU_676	Netarts WCSH inflow	0.318	0.007
OTU_2179	Netarts WCSH inflow	0.316	0.006
OTU_27	Netarts WCSH inflow	0.314	0.003
OTU_1735	Netarts WCSH inflow	0.313	0.024
OTU_1947	Netarts WCSH inflow	0.311	0.013

OTU_28	Netarts WCSH inflow	0.308	0.026
OTU_2143	Netarts WCSH inflow	0.305	0.01
OTU_2416	Netarts WCSH inflow	0.303	0.008
OTU_2208	Netarts WCSH inflow	0.298	0.006
OTU_1561	Netarts WCSH inflow	0.286	0.037
OTU_626	Netarts WCSH inflow	0.285	0.013
OTU_1871	Netarts WCSH inflow	0.281	0.027
OTU_2249	Netarts WCSH inflow	0.281	0.003
OTU_1671	Netarts WCSH inflow	0.276	0.007
OTU_42	Netarts WCSH inflow	0.275	0.011
OTU_2086	Netarts WCSH inflow	0.275	0.018
OTU_1398	Netarts WCSH inflow	0.271	0.008
OTU_2319	Netarts WCSH inflow	0.262	0.014
OTU_1727	Netarts WCSH inflow	0.261	0.006
OTU_60	Netarts WCSH inflow	0.257	0.005
OTU_1854	Netarts WCSH inflow	0.248	0.011
OTU_2339	Netarts WCSH inflow	0.243	0.024
OTU_2318	Netarts WCSH inflow	0.238	0.03
OTU_33	Netarts WCSH inflow	0.232	0.018
OTU_23	Netarts WCSH inflow	0.232	0.026
OTU_1512	Netarts WCSH inflow	0.228	0.007
OTU_1043	Netarts WCSH inflow	0.203	0.036
OTU_1621	Netarts WCSH inflow	0.200	0.045
OTU_891	Netarts WCSH inflow	0.198	0.032
OTU_162	Netarts WCSH inflow	0.197	0.023
OTU_68	Netarts WCSH inflow	0.184	0.038
OTU_2258	Netarts WCSH inflow	0.140	0.026
OTU_2340	Netarts WCSH inflow	0.135	0.045
OTU_30	Yaquina SW	0.557	0.001
OTU_1378	Yaquina SW	0.524	0.001
OTU_26	Yaquina SW	0.475	0.003
OTU_132	Yaquina SW	0.474	0.001
OTU_110	Yaquina SW	0.428	0.001
OTU_46	Yaquina SW	0.428	0.002
OTU_165	Yaquina SW	0.422	0.001
OTU_2233	Yaquina SW	0.406	0.002
OTU_2370	Yaquina SW	0.400	0.006
OTU_2326	Yaquina SW	0.383	0.001
OTU_744	Yaquina SW	0.378	0.001
OTU_277	Yaquina SW	0.377	0.001
OTU_1904	Yaquina SW	0.357	0.007
OTU_820	Yaquina SW	0.347	0.001
OTU_1373	Yaquina SW	0.346	0.002
OTU_1624	Yaquina SW	0.337	0.002
OTU_295	Yaquina SW	0.318	0.001
OTU_373	Yaquina SW	0.317	0.002
OTU_565	Yaquina SW	0.315	0.005
OTU_1907	Yaquina SW	0.311	0.014
OTU_783	Yaquina SW	0.310	0.007

OTU_88	Yaquina SW	0.297	0.003
OTU_1136	Yaquina SW	0.295	0.018
OTU_2215	Yaquina SW	0.295	0.005
OTU_24	Yaquina SW	0.289	0.045
OTU_1458	Yaquina SW	0.257	0.022
OTU_2237	Yaquina SW	0.242	0.049
OTU_2270	Yaquina SW	0.233	0.018
OTU_716	Yaquina SW	0.209	0.046
OTU_423	Yaquina SW	0.206	0.045
OTU_161	Yaquina SW	0.189	0.015
OTU_64	Yaquina SW	0.186	0.046
OTU_72	Yaquina SW	0.175	0.033
OTU_2335	Yaquina SW	0.154	0.038

Table S3: Average coefficient of variation (CV, 100% × standard deviation average^{-1}) for concentrations of total *Vibrio* spp., *V. coralliilyticus*, and total heterotrophic bacterial observed at different temporal and spatial scales in this study. Note that these CV were calculated from concentrations (cells mL^{-1}), whereas the regressions presented in Table 3 were performed using log-transformed concentrations.

		**Coefficient of Variance**		
**Location**	**Scale**	***Vibrio* spp. cells mL^{-1}**	***V. coralliilyticus* cells mL^{-1}**	**Heterotrophic bacteria cells mL^{-1}**
Netarts WCSH inflow	Biological replicates	55.8%	78.9%	7.8%
Netarts WCSH inflow	Within-day	55.4%	75.6%	23.4%
Netarts WCSH inflow	Among days	73.6%	176%	23.1%
Netarts tidal flat SW	Biological replicates	64.6%	76.9%	7.7%
Netarts tidal flat SW	Among stations	147%	170%	29.2%
